# Climate Change and Nutrition: Implications for the Eastern Mediterranean Region

**DOI:** 10.3390/ijerph192417086

**Published:** 2022-12-19

**Authors:** Ayoub Al-Jawaldeh, Maya Nabhani, Mandy Taktouk, Lara Nasreddine

**Affiliations:** 1 Regional Office for the Eastern Mediterranean (EMRO), World Health Organization (WHO), Cairo 7608, Egypt; 2 Faculty of Agricultural and Food Sciences, American University of Beirut, Beirut 11-0236, Lebanon; 3 Nutrition and Food Sciences Department, Faculty of Agriculture and Food Sciences, American University of Beirut, Beirut 11-0236, Lebanon

**Keywords:** climate change, nutrition, nutritional status, population, Eastern Mediterranean Region

## Abstract

The Eastern Mediterranean Region (EMR) is considered among the world’s most vulnerable to the dire impacts of climate change. This review paper aims at (1) characterizing climate change in countries of the EMR; (2) examining the potential effects of climate change on the nutritional and health status of the population; and (3) identifying the most vulnerable population groups. The paper explored several climate change indicators including daily temperatures, extreme temperature, daily precipitation, extreme precipitation (flooding, drought, storms, etc.), humidity, CO_2_ concentrations and sea surface temperature in EMR countries. Findings suggest that climate change will exert a significant adverse effect on water and food security and showed that the nutritional status of the population, which is already characterized by the triple burden of malnutrition, is likely to worsen via three main pathways mediated by climate change, namely, its impact on food security, care and health. Women, infants, children, those living in poor households and those experiencing displacement will be among the most vulnerable to the nutritional impacts of climate change. The paper concludes with a set of recommendations from the Initiative on Climate Action and Nutrition, which can support the region in tackling the critical nexus of climate change and nutrition.

## 1. Introduction

Climate change impacts almost every aspect of life. Rising global temperatures, shifts in precipitation patterns, and an increase in the intensity of heat waves and the frequency of extreme weather events are adversely affecting various systems that are integral for good nutrition, such as food production, dietary intake, health, social security and water/hygiene [1,2,3]. Nature-induced causes of climate change are exacerbated by human-led, environmentally damaging processes and actions, thus hauling the world into a “Code Red for Humanity” [4,5]. The 27th Conference of the Parties to the United Nations Framework Convention on Climate Change—COP27—held in November 2022—came at a time when the alarm was raised against the insufficiency of climate finance, undelivered pledges and a rapidly closing window of opportunity for adaptation and action [6]. In order to highlight the urgency and help increase the momentum for action that will address the critical nexus of climate change and nutrition, the COP27 Presidency is developing, along with several UN agencies and partners, a global multisectoral initiative on climate action and nutrition (I-CAN) that will help foster collaboration between countries at the global and regional levels [1].

The Eastern Mediterranean Region (EMR) of the World Health Organization (WHO) comprises 22 countries spread over East/North Africa and South/West Asia, with an estimated population size of 745 million [7]. The region is highly diverse, and great social and income level disparities exist between its countries [8]. Countries of the EMR may in fact be divided into two main groups based on their Gross National Income (GNI): the oil-producing high-income countries (HIC), and the low and medium-income countries (LMIC), with variations in per capita annual gross domestic products (GDP) ranging from only USD 445 in Somalia to more than USD 36,000 in the oil-rich Arab Gulf States in 2021 [9]. Countries of the EMR are among the world’s most vulnerable to the dire impacts of climate change [10]. On the one hand, the region already suffers from extreme fluctuations in temperatures and precipitation, as well as natural water and agricultural land scarcity. It is predicted that these patterns will more likely worsen in the coming years, with climate change, leading to a further reduction in precipitation levels of 15–45% by the end of the century [11], with intense pressure on agriculture; the great majority of which (70%) is rain-fed [12]. On the other hand, trends in the region predict a doubling in population size in the coming decades [11], thus compounding the effect of climate change with additional significant challenges to livelihoods and food security [13]. The region already harbors a high prevalence of food insecurity and a double burden of malnutrition characterized by the co-existence of undernutrition, micronutrient deficiencies, as well as overweight and obesity [14]. Therefore it is hypothesized that the prevalence of food insecurity and the burden of malnutrition will be further exacerbated as climate change continues to unfold in the region [14].

More vulnerable communities will be at a higher risk of food insecurity, accelerating pernicious consequences on productivity, migration patterns, nutrition and health [15,16]. To better identify the region’s vulnerabilities and to evaluate the countries’ capacities to address the critical nexus of climate change and nutrition, this review paper aims at (1) characterizing climate change in countries of the EMR based on available indicators; (2) examine the potential effects of climate change on the nutritional and health status of the population; and (3) identify the most vulnerable population groups to these effects.

## 2. Approach

A comprehensive literature review was conducted, including reports, individual studies and review articles that reported on climate change-related indicators in countries of the region, as well as the association of climate change with food security, food production/availability, food access or nutritional indicators. Electronic databases (MedLine, PubMed, Scopus and Google Scholar) were searched between 4 November 2022 and 14 November 2022. Keywords that were used included climate, climate change, temperature, precipitation, rainfall, flood, drought, storm, landslide, humidity, CO_2_ concentration, CO_2_ emission, health, nutrition, food security, food production, food availability, food access and Eastern Mediterranean Region (and individual countries within the EMR). The following United Nations (UN) databases were also consulted: WHO/United Nations Framework Convention on Climate Change (UNFCCC) and the United Nations Development Programme (UNDP) Country Climate Change Profiles. Data related to climate change indicators were retrieved for the various countries of the region. 

## 3. Findings and Discussion

### 3.1. Climate Change in Countries of the EMR

#### 3.1.1. Indicators of Climate Change in Countries of the EMR

Although historically, countries of the EMR have contributed relatively little to Greenhouse Gas (GHG) emissions and hence to the instigation of climate change, the region is among the world’s most vulnerable to climate change, affected by rising temperatures, insufficient and decreasing rainfall, frequent sand and dust storms, intensified droughts, and flooding [3]. The region includes several countries that are ranked among the most vulnerable nations to climate change worldwide. These include Afghanistan (ranked 176th out of 181 in the 2019 Notre Dame Global Adaptation Initiative, ND-GAIN index [17,18]), Djibouti (ranked 117 out of 181 in the 2019 ND-GAIN index [18,19]), Pakistan (ranked 152 out of 181 in the 2019 ND-GAIN index) [17,18] and Iraq (classified as the fifth-most vulnerable country to climate breakdown [20]).

**Figure 1:**
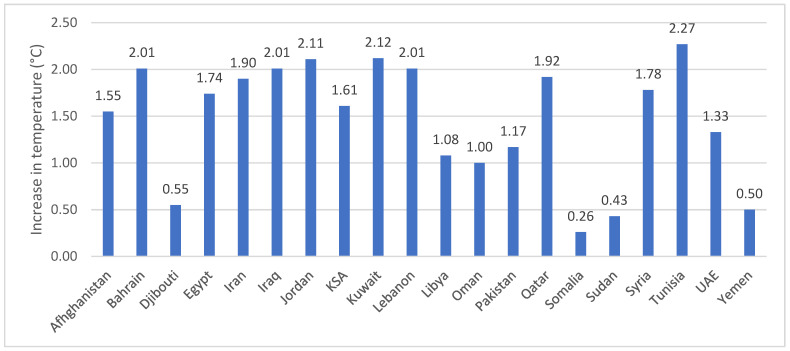
Increase in temperatures in various countries of the EMR between 1901 and 2021. Data retrieved from the World Bank Climate Change Knowledge Portal [21,22,23,24,25,26,27,28,29,30,31,32,33,34,35,36,37,38,39,40]. Abbreviations: EMR: Eastern Mediterranean Region; KSA: Kingdom of Saudi Arabia; UAE: United Arab Emirates.

Available data on several climate change indicators are presented in Supplementary Table S1 for the 22 countries of the EMR. These indicators include daily temperatures, extreme temperature/temperature threshold, daily precipitation, extreme precipitation (flooding, drought, storms, etc.), humidity and CO_2_ concentrations. As shown in Figure 1, available data document increases in average temperatures between 1901 and 2021 across various countries of the region, whereby the increase was lowest in Somalia (0.26 °C) and the highest in Tunisia (2.27 °C) [21,22,23,24,25,26,27,28,29,30,31,32,33,34,35,36,37,38,39,40]. Data on extreme temperature/temperature thresholds also show a similar increasing trend in the annual maximum temperature in various countries of the region (Supplementary Table S1). The Regional Initiative for the Assessment of Climate Change Impacts on Water Resources and Socio-Economic Vulnerability in the Arab Region (RICCAR) has released a recent technical report [41] that used climate modeling and analysis to produce climate projections for the Mashreq region (which covers many EMR countries in Western Asia and Northeast Africa). Projected climate changes were conducted for several indices including temperature, for the near future period 2021–2040 as well as the mid-century period 2041–2060. All the projections showed an increase in temperature over the region [41], and the magnitude of the increase was similar to that reported by an earlier report on the Arab region, where the general increase in temperature was estimated at 1.2–1.9 °C at mid-century and 1.5–2.3 °C by end-century [42]. The projections in extreme events also showed an increase in the annual number of hot days (where maximum daily temperature > 35 °C), as well as the annual number of tropical nights (where minimum daily temperature > 20 °C) throughout the Mashreq region [41]. 

**Figure 2:**
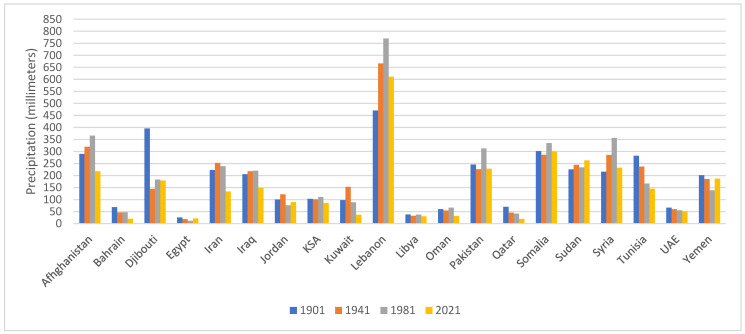
Trend in annual precipitation (millimeters/year) in various countries of the EMR. Data retrieved from the World Bank Climate Change Knowledge Portal [21,22,23,24,25,26,27,28,29,30,31,32,33,34,35,36,37,38,39,40]. Abbreviations: EMR: Eastern Mediterranean Region; KSA: Kingdom of Saudi Arabia; UAE: United Arab Emirates.

Data on average daily precipitation rates in various countries of the region are illustrated in Figure 2. A decreasing trend was observed over time in several countries including Afghanistan, Bahrain, Djibouti, Iran, Iraq and Kuwait, while the trend was less clear in other countries. RICCAR projections related to precipitation rates also showed variability between countries but the projections documented a consensus for reduced wintertime precipitation by mid-century across the relatively water-rich areas of the Mediterranean coast and mountains [41]. Projections also showed increased summertime precipitation over the dryland or desert areas [41], while cautioning that these increases are quite small and do not carry much impact on the overall water cycle in these already dry areas [41]. Projections related to the annual number of 10 mm precipitation days showed decreasing trends over time and similar results were obtained for the annual number of 20 mm precipitation days for the end of the century, suggesting a projected overall decrease in rainy days with such intensities over the region [42].

Data on extreme precipitation indicators (e.g., drought, floods and storms) are presented in Supplementary Table S1. In brief, there has been an increase in the severity of droughts in several countries of the region. For instance, in Afghanistan, drought severity has significantly increased between 1901 and 2010, in the southern provinces of Kandahar, Helmand and Nimruz during the wheat growing season (i.e., November to May), while drought severity during the rice and corn growing seasons (i.e., July to September) intensified significantly in the western part of the country [17,43]. Severe drought events such as that of 2011 have pushed millions of people into food insecurity and poverty in Afghanistan [17]. Djibouti has also experienced notable increases in aridity and droughts intensity, the most significant occurring in 1989, 1994, 2004 and 2005 [19,44]. Similarly, Tunisia has been experiencing increased periods of drought and dry spells, which were observed in 1982, 1987 to 1989 and 1993 to 1995, with its most severe drought in over 50 years taking place from 1999 to 2002 [45,46,47]. Predicted environmental changes in the region depict higher temperatures, more frequent and stronger heat waves and droughts [14,48,49,50].

Floods are also an increasing concern in countries of the region. For instance, in Morocco, the patterns of seasonal rainfall have shifted to longer and more intense rain events (namely, in October and November), which are often leading to floods [51]. It was also estimated that 18.2% of the land area of Qatar is increasingly susceptible to inland flooding [52]. The Kingdom of Saudi Arabia (KSA) experienced five major floods between 2018 and 2020, which resulted in property destruction and significant loss of plant species [53]. Recent years have witnessed stronger precipitation events resulting in flash flooding in Djibouti, Iraq and Tunisia [19,20,45,46,54]. Reports from Egypt and Iran indicate that the possibility of severe floods will continue to increase in the future because of climate change [55,56].

**Figure 3:**
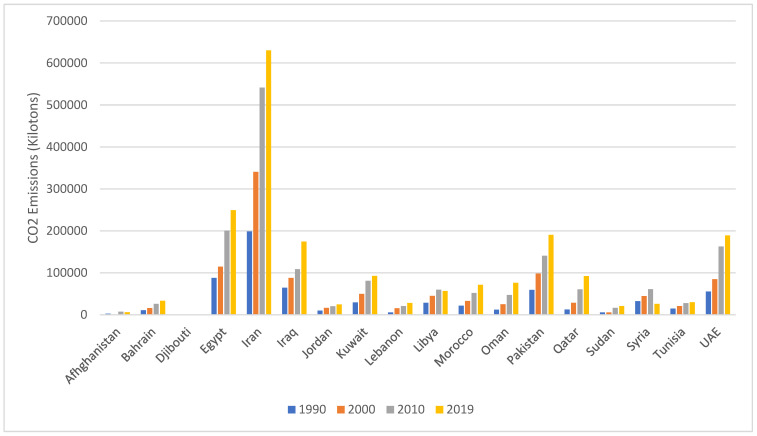
Trend in CO_2_ emission (kilotons) over time in countries of the EMR. Data retrieved from the World Bank [9,19,61,62,63,64,65,66,67,68,69,70,71,72,73,74,75,76]. Abbreviations: EMR: Eastern Mediterranean Region; UAE: United Arab Emirates.

As shown in Figure 3, and similar to observations from other parts of the world [57,58,59,60], CO_2_ concentrations/emissions have been following an increasing trend over time in the EMR. Iran has the highest level of CO_2_ emissions in the region, reaching 630,010 kilotons in 2019. The relative increases in CO_2_ emissions (calculated as (2019 levels/1990 levels) × 100) ranged between +79% in Syria and +733% in Qatar, with a regional average of 327%.

In addition to the indicators listed in Supplementary Table S1, sea surface temperature is also classified as a climate change index. Data on this indicator are very limited in the region. In Jordan, a significant long-term increase in temperature of about 0.03 °C per year was observed in the lower layer (200–1000 m in depth) since 1989 [77]. In Oman, summer average sea temperatures have increased since 1960 by over 2 °C at the sea surface and by approximately 1 °C at a depth of 300 m [78]. 

#### 3.1.2. Climate Change and Water Security in the EMR

Climate change is predicted to amplify the challenges and constraints to the task of ensuring adequate water supply necessary to meet the region’s growing needs, and is likely to continue to reduce total and per capita water availability to dangerously low levels [16]. Countries of the EMR are predicted to be among the first in the world to “essentially run out of water” [79]. The arid to semi-arid region is already notoriously known for its water scarcity and water stress due to its competing use in agriculture, industries and by rapid population growth, such that water supplies are being depleted faster than they are being replaced by precipitation [80,81]. Estimates indicate that countries in the EMR have the highest losses of freshwater as compared to other countries worldwide [81], as the region suffers from groundwater depletion, coupled with high rates of extraction and use of both ground and surface water [82]. The list of the 20 most water-stressed countries worldwide includes 14 EMR countries, namely, Kuwait, United Arab Emirates (UAE), KSA, Libya, Qatar, Yemen, Egypt, Bahrain, Syria, Sudan, Oman and Pakistan. Jordan and Tunisia [83] (Table 1).

**Figure 4:**
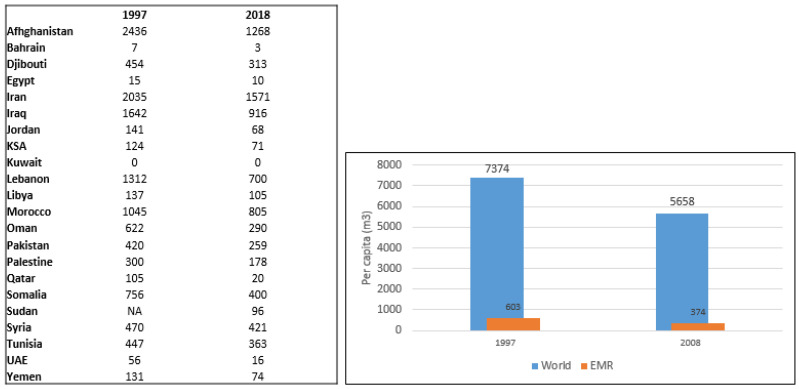
Per capita levels of renewable internal freshwater resources (m^3^) in countries of the EMR and change in these levels in the world vs. in the EMR. Abbreviations: EMR: Eastern Mediterranean Region; KSA: Kingdom of Saudi Arabia; UAE: United Arab Emirates.

Some models anticipate that by 2025, between 80 and 90 million people in the region will experience some sort of water stress [14,84,85], particularly in low-lying coastal parts of the EMR, where decreasing levels of precipitation and the inability of countries to replenish internal fresh water supplies (rivers and aquifers) are coupled with rising sea levels due to climate change [11,14,86]. Data on per capita levels of renewable internal freshwater resources are shown in Figure 4 for the years 1997 and 2018. Importantly, compared to the world’s per capita levels of renewable internal freshwater resources, which have decreased from 7374 m^3^ in 1997 to 5658 m^3^ in 2018 (i.e., percent change of −23%), the decrease in per capita renewable internal freshwater resources was sharper in the EMR, estimated at −38% (from 603 m^3^ in 1997 to 374 m^3^ in 2018).

The intensification of the water crisis will have its toll on the already insufficient food production and the fragile food security in the region [81]. Water demand for other sectors in the region is also projected to increase with climate change [87,88], thus amplifying the effect of water scarcity in the agricultural sector, and consequently on food security [81].

Increased water stress/flooding that accompanies climate change will also contribute to decreased water quality and hygiene, which in turn will contribute to an increased risk of waterborne diseases, and diarrheal diseases in particular [89,90]. Poor access to safe drinking water is already a challenge for many countries of the region [91]. On average, 13% (84.4 million people) of the region’s inhabitants do not have access to basic water services, with higher prevalence (64.6 million) in countries such as Afghanistan, Pakistan, Sudan, Somalia, and Yemen and to a lower extent (15 million) in Iran, Iraq and Morocco. While for the remaining 87%, basic drinking water is accessible, management of its safe delivery does not always adhere to Sustainable Development Goals (SDGs) targets [91,92].

#### 3.1.3. Climate Change and Nutrition

The EMR harbors a triple burden of malnutrition, with undernutrition and micronutrient deficiencies coexisting with overnutrition in most countries [93]. In 2018, the prevalence of stunting, wasting and underweight in the region was estimated at 28%, 8.69% and 18%, respectively, with the highest burden of stunting (>30%) existing in Afghanistan, Djibouti, Pakistan, Sudan and Yemen [93]. A wide range of anemia prevalence was observed among countries of the EMR, such that ranges of 7.4% to 88% were reported in children aged <5 years and of 19.9% to 63% in women of childbearing age [93]. The rising prevalence of overweight and obesity in the region, among both adults and children, is alarming. Overweight prevalence in the region was estimated to be 27% among adults and 16.5% among school-aged children, while the prevalence of obesity was 24% in adults and 4.8% in school-aged children, with HICs, such as Bahrain, Kuwait, Qatar and the UAE reporting the highest levels of obesity [93]. Available evidence suggests that the nutritional status of the population in the region is expected to worsen with the ongoing climate change [89].

The link between nutrition and climate change is intricate, operating through various routes and multiple directions. The WHO technical series on Adapting to Climate Sensitive Health Impacts-Undernutrition established several links between climate change indicators and risk factors for malnutrition [89,94]. These are summarized in Table 2.

Several conceptual frameworks have been developed to present these intricate pathways [89,95,96,97], the majority of which were based on the United Nations International Children’s Emergency Fund (UNICEF) conceptual framework for undernutrition [89]. Essentially, these conceptual frameworks propose that the impacts of climate change on nutrition outcomes are primarily mediated via three different causal pathways, namely, food security, care and health [89,94]. These are reviewed below, with a contextualization to the EMR.

### 3.2. The Food Security Pathway

Climate change is predicted to adversely affect all aspects of food security (availability, access, utilization and stability) in the EMR, a region that already harbors a high burden of food insecurity. In 2015, Food and Agriculture Organization (FAO) reported that, as compared to other regions worldwide, the EMR is the only region experiencing decreased food security with “a serious setback in its fight against hunger” [98]. More than 116 M people (27.2%) suffer from food insecurity in the region [99], with severe food security prevalence estimated at 11.8% in 2016, thus placing it among the most food insecure regions in the world [100]. Food insecurity prevalence in the region is ascribed to heavy dependence on food imports combined with low agricultural production, social inequalities and unstable political situations [101], with complex crises in the region, such as in Iraq, Syria and Yemen, exacerbating the situation [102]. This was further intensified by the COVID-19 pandemic and its impact on livelihoods and food security [99].

The EMR countries are diverse, not only in their economic structures, but also in their overall food security, and their ability to address their current and future food security challenges [102,103]. For example, variations in food security exist between HIC and LMIC in the region. The Gulf Cooperation Council (GCC) population is less likely to experience food insecurity (8%), while those in middle and low-income levels are exposed to higher rates of food insecurity (25–40%), with higher prevalence seen among populations in conflict-affected countries [99]. The growing population in the region is an additional strain on food and water security. For instance, it was estimated that the population of the Arab countries will almost double by 2050—from 357 million residents to 646 million [104], with an annual population growth of 1.84% [105]. The impact on food security in the region will be manifested through additional demands on energy, higher demand for water-intensive animal food products and increased industrialization.

Climate change is expected to affect food security in all its parameters (availability, accessibility, utilization and stability) in the EMR region, particularly LMICs, such that not only will food production not meet demand, but communities will be faced with challenges to consistently have access to safe and nutritious food in the right quantity [15,98,106,107,108].

#### 3.2.1. Availability

Climate change has a direct effect on food availability in the region. Faced with scarce arid land and water resources, the ability of EMR countries to produce food is further compromised by climate change [13,15,107]. The region’s increasing temperature and sea levels, coupled with rising land salinity and shift in species, are expected to lead to environmental changes, which will negatively impact marine life, crops and ultimately food production [109]. With rising temperatures, new pests and disease vectors will proliferate and expose crops, livestock and fish to new diseases [109]. For example, in 2019, the Middle East was exposed to a desert locust outbreak, which was instigated by unusual weather conditions aggravated by climate change, thus destroying food and vegetation in the region and threatening food security [94].

Agriculture, irrigation water requirements and livelihood will be significantly impacted by long-term warming, increasing atmospheric carbon dioxide (CO_2_) and changing rainfall patterns [110,111,112]. The impact will be more severe in areas that are dependent on rain-fed agriculture, such as countries of the EMR [12]. Weather-dependent rain-fed regions make up 80% of cultivated land and 100% of pastureland; therefore, changes in climate will most likely negatively affect yields, as well as the quality and diversity of both wild and cultivated food products [89]. Moreover, increased fluctuations between climate extremes, such as intensified and consecutive drought, increased length, frequency or intensity of heat waves, and increased frequency of heavy precipitation and flooding may exert greater adverse impacts on crop yields than average climate change [111]. The impact of climate change, on the production of four main crops in Iran (wheat, barley, rice and corn) and on water productivity, in the Zayandeh-Rud River Basin, was evaluated by Gohari et al. [113]. The study revealed that the production of crops with higher demand for irrigation water (rice and corn) may be compromised due to their higher vulnerability to climate change. Using various modeling scenarios, studies conducted in Egypt, Jordan, KSA and Pakistan have documented a dose–response relationship between temperature increases and decreased crop yield for grains, olives and vegetables [114,115,116,117]. For instance, a fixed effect regression framework implemented in a study from KSA suggested that a one-degree Celsius increase in temperature would be associated with a reduction in crop yields by 7–25% [118]. A study conducted in Pakistan reported that an increase in temperature by 3 °C in 2050, will decrease the per capita availability of wheat to 84 kg per year, compared to 198 kg per year in 2012, after taking into consideration climate change scenarios and population growth in the coming years [119]. Agricultural biodiversity would also be negatively impacted by climate change. This loss of biodiversity, in turn, diminishes food systems’ capacities to respond to external and internal stressors, including additional climate change [120]. Taken together, these factors will have direct repercussions on the production and availability of foods in countries of the region and ultimately influence the availability of sufficient food for households and individuals [89]. 

Another effect of climate change on food availability is mediated via its impact on food safety and quality (and hence the availability of safe and nutritious foods). Food safety and quality may in fact be adversely affected by warmer ambient temperatures, which promote microbial growth, and enhance spoilage and transmission of food-borne illnesses [89].

#### 3.2.2. Accessibility

The insufficient food production in countries of the region has been historically counterbalanced by importing food through international markets; however, the extreme fluctuations of global prices, in recent years, represent heightened risks in food insecurity for heavily populated LMICs with lower capacity to respond to price shocks [13,15]. The World Bank (2013) estimates that countries in the EMR import around 50% of their wheat and barley, 40% of their rice and 70% of their maize, with some Arab countries relying totally on import when it comes to their cereal caloric needs [121]. In Egypt, a large portion of the food supplies (wheat, cereals, vegetable oil and sugar) that are regularly consumed by low-socioeconomic groups is imported [122]. Libya and Morocco also rely heavily on imports to cover their cereal consumption requirements (for both human consumption as well as feed) [123,124]. Based on the vulnerability index for key food commodities [125], Jordan and Kuwait ranked as having severe vulnerability for grains and oilseeds [125], while Qatar was assigned medium vulnerability for meat and milk, high vulnerability for grains and oil-seeds and severe vulnerability for rice [125]. Such heavy reliance on food imports in the EMR contributes to food insecurity through exposure risks to the supply chain and fluctuations in prices, as seen during the global food crises in 2007/2008, 2010/2011 [98], and more recently during the Ukraine crisis, where the prices of key staple foods, for most families in the region, have dramatically risen (in Yemen and Syria, vegetable oil prices have risen by 36% and 39%, respectively; and in Lebanon, Libya and Palestine, wheat flour prices have risen 47%, 15% and 14%, respectively) [126].

Modeling studies related to climate change’s impact on food prices suggest that when adjusted to speculated inflation trends, the prices of staple foods, such as wheat, rice and maize could witness an increase of 31–106% by the year 2050 [127]. The increased food prices will adversely impact the accessibility of vulnerable communities to food in countries of the region [128,129]. In general, the growing population of urban consumers in countries of the EMR [130] are the most impacted since they are highly dependent on staple foods and have limited ability to participate in farming activities to meet their needs [110,131]. Resource-poor households usually adapt to their reduced access to food and increased prices by prioritizing calorie-dense but nutrient-poor foods in their diet [132].

#### 3.2.3. Utilization

Climate change can have a significant impact on nutrient absorption, diet quality and safety, and hence will impact food utilization [107]. Diarrheal episodes, which have been reported to reduce the uptake of nutrients, are linked to climate-sensitive pathways; the latter being associated with poor water quality, higher rates of food-borne illnesses and the higher possibility of their spread via direct contact. These risks are further intensified by the lack of access to clean and adequate water and poor hygiene [133]. In a systematic review conducted in 2015 on climate change in relation to health in the EMR, Khader et al. identified several studies [134,135,136,137,138,139,140] that have associated diarrheal diseases with climate change in countries of the region. In general, studies suggest that Pakistan is an endemic country that necessitates consistent monitoring, predominantly during flood seasons, especially as an unexpectedly high prevalence of Escherichia coli pathotypes has been reported in the country. Egypt, Tunisia and Jordan have also reported a high incidence of diarrheal diseases, particularly during summer [134,135,136,137,138,139,140].

Changes in temperatures, rainfall patterns and increased humidity can also alter the type and range of aflatoxin-producing fungi, which in turn can contaminate crops during cultivation, as well as postharvest and increase the population’s exposure to these toxins [141,142,143]. Similarly, variations in sea surface temperatures can affect marine life, with increased levels of poisonous toxins and Vibrios observed in warmer water. These toxins are absorbed in fish and shellfish, thus exposing ciguatera and other marine products to higher risks of poisoning [144,145].

Climate-related changes affecting food safety (including animal and vector behaviors, and altered pathogen, organism and pest livelihood, proliferation and transmission behaviors [146]) are more likely to bear severe adverse impacts in countries where food monitoring and surveillance systems are less robust. The majority of countries of the EMR, especially LMIC, have poor governance and policies around food safety [147], which further exacerbates the risk to public health and nutrition associated with acute and chronic exposure to contaminants [94].

While higher levels of CO_2_ in the environment may lead to higher plant mass, they may reduce protein synthesis in crops, such as wheat, soybeans and rice, by displacing nitrogen. Increased atmospheric CO_2_ may also decrease the content of beneficial minerals in some crops [110,148,149]. Available evidence indicates that the change in climate and the higher levels of atmospheric CO_2_ may lead to decreases in the levels of iron and zinc in crops, predominantly cereals and legumes [150]. For instance, it has been projected that increases in atmospheric CO_2_ will lead to a decreased growth in the global availability of nutrients, by 13.6% for iron, 14.6% for zinc as well as 19.5% for protein, by 2050 [151]. This may lead to an increased risk of zinc, iron and protein deficiencies among the populations of the EMR, whose main source of protein and micronutrients (zinc and iron) uptake depends on these crops, particularly in LMIC [152,153].

Food use and consumption patterns are also likely to shift with the changes in the availability of various cultivated or local food varieties, and the introduction of new varieties as adaptation strategies [89]. This may lead to increased consumption of high-energy, low-nutrient imported food products, thus reducing diet quality [94].

#### 3.2.4. Stability

Food is delivered to populations, locally and globally, through an intricate food system that includes all activities extending between production and utilization, such as transport, trade, storage, processing, packing, sale, consumption and disposal of food. Each of these components is vulnerable to the risks of climate change; thus, a particular food system is as volatile as the number of these elements from which it is comprised [154]. There is increasing evidence of the negative impact that climate change and extreme weather events, in particular, exert on the stability of the food system [110].

In a region where food security is already threatened [98], the sustainability of the food system plays a direct role in people’s access to affordable, safe and nutritious diets. It is expected that with desertification and decreased ability of food production, higher dependence on food import will prevail, making the region more susceptible to global market shocks [155,156]. As food production decreases and food prices increase, supply disruptions will intensify and the sustainability of the region’s food system will be further impacted, therefore increasing the risk of poor health and diet-related diseases among inhabitants of the EMR, especially in LMICs [4]. The increasing occurrence of droughts and floods in the EMR also constitutes a growing threat to food stability, thus leading to both chronic and transitory food and nutrition insecurity [157]. It has been proposed that in the long term, the recurrence of extreme events in the region can immensely undermine regional and local food systems [109,112], thus potentially triggering food crises and acute malnutrition and hunger [89].

### 3.3. The Care Pathway

Climate change was also suggested to affect appropriate care and feeding practices, such that access to health services and the quality of infant and young child care will also be negatively impacted by fluctuations in price, in addition to the adverse consequences to household food access and diet quality [158]. The negative impacts on care and on infant and young child feeding practices may be mainly mediated via increased maternal stress, interruptions to the support of infant feeding, as well as less access to clean water, sanitation and hygiene [94].

Climate-related impacts on food security, and hence on dietary diversity, also carry significant implications related to the caregivers’ ability to provide appropriately diverse foods for infants and young children as well as for themselves [94]. Data from 19 countries show evidence of significant correlations between dietary diversity in children, and temperature and rainfall levels [94]. It has also been suggested that breastfeeding practices may be negatively affected by climate change. More specifically, a mother’s ability to breastfeed may be compromised by heat stress or dehydration, in addition to the fact that climate-related impacts on a maternal diet may also influence breastmilk quality [94]. Additional negative impacts on breastfeeding practices in the region have been related to climate-forced migration, leading to disruptions to breastfeeding [159] and an increase in using substitutes for breast milk, thus negatively affecting infant nutrition and health. Although these pathways are based on a logical framework, limited data currently exist to support many of them [94].

### 3.4. The Health Pathway

The health of inhabitants of the EMR is among the worst impacted by climate change (second only to Africa); even though historically, GHG emissions in countries of the region have been relatively low, and thus have contributed little to the onset of climate change [160]. Adverse health outcomes related to climate change have been documented in a systematic review of research from EMR countries [3]. Among those commonly identified across the region are increases in water, food and vector-borne diseases (dengue, malaria, schistosomiasis and zoonotic cutaneous leishmaniasis), under-nutrition, heat-induced morbidity, cardiovascular and respiratory illnesses, rise in mental health issues, as well as allergic reactions and pulmonary diseases due to dust storms [161]. In light of the possible threats of climate change, many of these health-related challenges will most likely be exacerbated in the coming years.

Many water-related diseases linked to high temperatures, poor sanitation and contaminated water such as cholera and trachoma are expected to increase, as water security and access to sanitation are impacted by climate change [11]. The incidence rate of Cholera, in light of climate change, has been examined in several studies among countries of the region, whereby results showed a significant correlation with less rainfall and elevated temperatures and humidity [162,163,164]. The incidence of diarrheal diseases, which is the leading cause of malnutrition in children under five years of age [165], is expected to increase. Diarrhoeal disease and malnutrition are interrelated. On the one hand, malnutrition increases the burden of disease, as it is reported as a fundamental risk factor for diarrhoeal disease transmission. For example, extremely undernourished children are at a higher risk (more than nine-fold) of mortality [166]. On the other hand, decreased appetite and lower nutrient uptake coupled with increased metabolism, which is associated with diarrheal episodes, often lead to malnutrition. Moreover, undernutrition increases the duration, severity and mortality risk from other illnesses, such as acute respiratory infections, measles and malaria [89]. Similarly, climate variations favoring higher temperatures may exacerbate vector-transmitted diseases such as malaria and other parasites, as these are expected to proliferate better with higher temperatures [167,168,169]. A recent review examining climate change in relation to malaria indicated that, while considerable evidence shows that warmer conditions tend to facilitate malaria transmission, the relationship between rainfall and malaria may be nonlinear [170]. For instance, while the time between rainfall episodes can foster the rapid growth of mosquito populations, longer spacing between rainfall episodes can restrict the growth rate of mosquito populations. It also argued that, in some circumstances, drought can further promote vector breeding sites when the water pools in dried-out rivers and dams [170]. For example, in Venezuela, malaria mortality was shown to be more strongly associated with drought in the year preceding the outbreaks than with rainfall during epidemic years [170]. The EMR region already harbors a high burden of malaria, whereby 44% of the EMR inhabitants were estimated to live in areas where a high risk of malaria transmission prevails [171]. These areas include several countries including Afghanistan, Djibouti, Pakistan, Somalia, Sudan and Yemen [171], which face a high risk of malaria transmission and are threatened by epidemics and/or complex emergency situations [172].

It is predicted that undernutrition will be the biggest climate change-related health threat, with its most substantial impact foreseen in Asia and Africa (which harbor EMR countries), where the prevalence of childhood undernutrition and related morbidity and mortality is projected to increase [94]. Strong evidence exists that links increasing temperatures with a greater risk of pre-term and low birth weight deliveries [173,174]. This may be attributed to a variety of mechanisms, including maternal dehydration-related complications such as premature labor or the early rupture of membranes and impaired fetal growth as a result of reduced uterine blood flow [175]. The regional average prevalence of low birth weight is already high, estimated at 19.3% in 2018 [93]. Low birth weight (LBW) prevalence was found to exceed 30% in Sudan [176] and Pakistan [176] and reached as high as 45% in Yemen [177]. Alarmingly, an increasing trend in LBW prevalence was observed in Yemen [176,177], Pakistan [176], Lebanon [176], Oman [178,179], Somalia [180,181] and the Syrian Arab Republic [176]. A systematic review, by Phalkey et al. [182], has also suggested a link between climate change and stunting, with a significant correlation between childhood stunting at the household level and climate/weather fluctuations (e.g., precipitation, extreme weather events—floods/droughts—seasonality and temperature). The majority of the studies included in the review reported a significant association between worsening nutritional status among children, particularly stunting, and exposure to one or more climate/weather variables [182].

In combination with the above, emerging evidence suggests an adverse impact of climate change on the prevalence of diet-related non-communicable diseases due to an increased risk of overweight and obesity [94]. Paradoxically, while on the one hand, severe food insecurity and hunger are correlated with lower obesity prevalence, on the other hand, mild to moderate food insecurity is correlated with higher obesity prevalence, particularly where calorie-dense, commercially processed foods prevail at a low cost [183]. Price fluctuations of fresh foods can further exacerbate the situation by reinforcing dependency on high-energy processed foods, especially in the context of targeted marketing [184]. This is of particular concern to the EMR, where over the last thirty years, many of its countries have experienced economic development, which, coupled with rapid population growth [5], has led to the westernization of diets and increased reliance on processed foods [185]. In the HICs of the EMR, climate change and rapid development and urbanization are expected to further decrease fruit and vegetable intake, with a simultaneous increased demand for processed foods, thus further heightening the risk of non-communicable diseases (NCDs) and related deaths [186]. It is likely that these and other pathways leading to climate change-induced food and nutrition insecurity will exacerbate already-changing dietary patterns, which tend to be increasingly comprised of high-energy foods [94].

### 3.5. Identifying the Most Vulnerable Population Groups to the Impact of Climate Change on Nutrition

The impacts of climate change will have disproportionate effects, on those who, for physiological/demographic reasons, are at a higher risk for undernutrition. As such, women, infants and children would be among the most vulnerable population groups to the climate effects on nutrition [94].

The current nutritional status of women of reproductive age in the EMR is characterized by the coexistence of various forms of malnutrition including undernutrition, micronutrient deficiencies and overweight/obesity [93]. Women are at a higher risk of malnutrition given their higher needs for water food and nutrients to support critical stages of the lifecycle such as pregnancy and lactation [93,187]. Women are also more vulnerable to climate change effects given the intersections between gender, social systems, power dynamics, and societal norms and expectations, which lead to an extremely different experience of climate change and its impacts, among women [188]. In the region, cultural perceptions of nutritional requirements, which, in turn, govern patterns of food distribution within households, differentiate between males and females of all age groups, consequently leading to gender-related differences in vulnerability [189,190,191,192,193]. Therefore, this highlights the relevance of gender analysis in relation to climate, health and nutrition [89].

The current nutritional status of under-five children in the EMR is also characterized by a double burden of malnutrition. According to the joint child malnutrition estimates 2017 (UNICEF-WHO-World Bank) [194], the EMR ranks second worldwide, after Africa, in terms of its prevalence of wasting and severe wasting, with a prevalence of 9.1% and 3.8%, respectively. According to these estimates, the prevalence of stunting in the EMR also exceeds the world average (25.1% vs. 22.9%). At the same time, the EMR suffers from a high burden of under-five overweight/obesity, ranking second worldwide, after the Americas, with a prevalence of 6.7% [194]. With climate change, poor water and diet quality, household food insecurity, poor sanitation, suboptimal care practices and the increase in parasitic infections and diarrheal diseases are among the factors that contribute to an increase in malnutrition in this age group [89,94]. The interrelationship between malnutrition and infection implies that with a higher incidence of undernutrition, a rise in childhood morbidity and death rates will be observed [94]. Malnutrition is one of the major determinants of health, and its impact transcends from one generation to the other. Its impacts are most prominent early in the lifecycle (fetuses, infants and young children), whereby nutrient requirements are highest throughout growth and development. Adequate nutrient intake is most critical during the first 1000 days of a child’s life to avoid irreversible health outcomes later in life, such as impaired mental and physical development [195], with consequences on economic productivity, reproductive performance, immunity and cardio-metabolic health [196].

Besides physiological/demographic reasons for vulnerability, there are social, economic, environmental or political factors that may increase vulnerability to the effects of climate change [89]. The climate crisis will be experienced differently among different population groups, depending, for example, on socio-economical or displaced/refugee status [94]. When the consequences of climate change are considered in the context of economic losses and impact on livelihood in the region, they portray a grim picture for vulnerable groups in the LMIC of the EMR, especially when the rural–urban divide and social inequality and instability are factored in [15]. Although the agricultural sector in the region produces less than 10% of total needs, it constitutes 22% of total employment. In addition, rural areas are home to 70% of the poor communities [197]. Disadvantaged rural communities will be subjected to more social and health-related inequities, since they are considered the least able to adapt to the impacts of climate change [99]. The direct effects of climate change, such as extreme temperatures and floods, indirect consequences leading to the loss of agricultural areas, and the slowing down of the agricultural sector in the EMR, imply more migration to urban areas [198]. In combination with other factors, such as the sluggish expansion of labor-intensive sectors and the accelerated population growth, this is expected to result in high unemployment within fast-growing cities [15], therefore amplifying the number of food insecure in the region, which, in turn, is a predisposition to malnutrition and diet-related NCDs [4]. Populations that are more likely to suffer from the effects of the climate crisis are those that have contributed the least to it (with the lowest carbon footprint per person) [94].

Almost half of the countries of EMR are experiencing natural and manmade conflicts, which constitute a major challenge for the region [199]. As a result, the EMR harbors a high proportion of displaced populations/refugees. Table 3 provides an overview of the numbers and proportions of refugees and internally displaced populations in some countries of the EMR. Displaced populations and refugees have severe vulnerability to the impact of climate change given their lack of resources, and disrupted access to services and assets [89]. This population’s limited access to basic assets, such as health care, food, water and sanitation will be further disrupted by the climate change crisis [89].

## 4. Conclusions

This review paper characterized climate change in countries of the region and its potential impacts on nutrition. It suggests that climate change will exert a significant adverse effect on water and food security in countries of the region, which already are among the most water-stressed worldwide, and which harbor a high prevalence of food insecurity. The review also showed that the nutritional status of the population, which is already characterized by a triple burden of malnutrition, is likely to worsen via three main pathways mediated by climate change, namely, its impact on food security, care and health. Within the population, women, infants, children, those living in poor households and those experiencing displacement will be among the most vulnerable to the nutritional impacts of climate change. The region faces a serious public health challenge with the further deterioration of nutritional status in its countries, and profound socioeconomic consequences would be expected to manifest, both at the individual and household levels [89]. The major vulnerabilities of the region vis a vis climate change and its adverse effects on nutrition are summarized in Box 1.

Box 1 **Key characteristics of the EMR that increase its vulnerability to climate change and its impact on nutrition. Abbreviations: EMR: Eastern Mediterranean Region.**

- The region already suffers from extreme fluctuations in temperatures, and precipitation.
- The region is characterized by high aridity and exposure to droughts in many of its countries, which limit food production and food availability.
- The region is among the most water-stressed worldwide.
- Countries of the region have low agricultural self-sufficiency.
- The region is characterized by high reliance on imports, particularly staple foods, which increases the countries’ susceptibility to global market shocks that accompany climate change, and affects the population’s access to sufficient, safe and nutritious food.
- The region harbors countries that are endemic for vector-borne diseases such as malaria, and hence are exposed to severe worsening of this endemic as ambient temperatures increase.
- The region is witnessing a significant demographic growth.
- The region harbors a high burden of food insecurity.
- The region is characterized by a high burden of malnutrition in all of its forms.
- The EMR has a high proportion of displaced populations/refugees.

Although this paper tackled the effect of climate change on nutrition, it is important to acknowledge that the relationship between climate change and nutrition is, in fact, bi-directional, whereby the foods and diets consumed by the population can also affect climate change [1]. Food systems impact the environment in various ways, such as the direct release of GHGs (methane, nitrous oxide and CO_2_) into the atmosphere, and land use, which leads to additional release of CO_2_, while further reducing carbon sinks. In addition, dietary choices drive various food production systems; therefore, resulting in varied emissions and environmental footprints [1]. The bidirectional correlation between nutrition and climate change indicates that sustainable, resilient and healthy diets constitute a significant link between nutrition and climate change [201], and are a necessary condition to good nutrition and a pre-requisite to tackling malnutrition, in all its forms, as well as an impetus of sustainable development [202].

## 5. Recommendations

In order to progress towards achieving their targets of Agenda 2030, particularly those of SDG13, SDG2 and SDG3 (climate action, eliminating hunger and health, respectively), countries will need to abandon the “business as usual” mode of operation, and proceed by addressing the intricate linkages between climate and nutrition in an integrated way. I-CAN has developed a set of objectives and outcomes, the ultimate purpose of which are to advocate for linking actions, in order to increase the momentum of progress in both climate (mitigation and adaptation) and nutrition; to describe deliverables capable of examining progress as an outcome of integrated action; and to enable lending technical and high-level support to various countries, so that they may achieve these deliverables. The outcomes are organized around four pillars: (1) implementation, action and support; (2) capacity building, data and knowledge transfer; (3) policy and strategy; and (4) investments. It is therefore recommended that the region adopts the priority action areas in each of these four pillars as summarized in Table 4. These recommendations can be spearheaded by the WHO Regional Office for the Eastern Mediterranean (EMRO), to support the Strategy on Nutrition for the Eastern Mediterranean Region 2020–2030, which was endorsed by the WHO Regional Committee for the Eastern Mediterranean in October 2019 [91]. It is only through concerted, multi-layered action that the delicate link between climate change and nutrition can be addressed.

## Figures and Tables

**Table 1:** Levels of water stress * in EMR countries in 2019 ordered from the most to least water stressed.

Country	Level of Water Stress (%)
Kuwait	3851
UAE	1631
KSA	974.2
Libya	817.1
Qatar	431
Yemen	169.8
Egypt	141.2
Bahrain	133.7
Syria	124.4
Sudan	118.7
Oman	116.7
Pakistan	108.6
Jordan	104.3
Tunisia	96
Iran	81.29
Iraq	79.51
Lebanon	58.79
Afghanistan	53.75
Morocco	50.75
Palestine	47.01
Somalia	24.53
Djibouti	6.33

* Water stress is defined as the amount of freshwater withdrawn out of the total available renewable freshwater resources to fulfill the needs of major economic sectors. This ratio takes into account environmental water requirements. Data retrieved from the Food and Agriculture Organization of the United Nations [83].

**Table 2:** Links between climate change indicators and risk factors for malnutrition.

Climate Change Indicator	Risk Factors for Malnutrition				
	Availability of SNS Food	Access to SNS (Economic or Physical)	Food Safety and Quality	Maternal and Child Care	Diarrheal Disease
Daily temperature	X	X	X	X	X
Extreme temperature	X	X	X	X	X
Daily precipitation	X		X		X
Extreme precipitation (floods drought, storms, etc.)	X	X	X	X	X
Sea-surface temperature	X		X		X
CO_2_ concentration			X		

Adapted from Bush et al., 2022, and World Health Organization, 2019 [89,94]. Abbreviations: SNS: Sufficient, nutritious and safe food.

**Table 3:** Numbers and proportions of refugees and internally displaced populations in some countries of the EMR.

Country	Population	Refugees	Internally Displaced Population	Displaced as Share of Population *
Libya	6,735,277	3141	160,456	2.4%
Iraq	43,533,592	280,072	1,206,530	3.4%
Sudan	45,657,201	1,103,918	3,260,522	9.6%
Yemen	32,981,641	89,467	4,299,575	13.3%
Somalia	17,065,581	13,804	2,967,500	17.5%
Lebanon	5,592,631	1,328,541	50	23.8%
Jordan	11,148,277	3,047,612	-	27.3%
Syria	21,324,367	589,542	6,661,640	34.0%
Palestine	5,133,392	2,400,208	11,711	47.0%
Total	189,171,959	8,856,305	18,567,984	14.5%

* Share = (refugees + internally displaced population)/Population. Reference: UNHCR’s Refugee Data Finder [200]. Abbreviations: EMR: Eastern Mediterranean Region.

**Table 4:** Outcomes and examples of action recommended by I-CAN. Adapted from I-CAN [1]. Abbreviations: EMR: Eastern Mediterranean Region; GIIN: Global Impact Investing Network; I-CAN: Initiative on Climate Action and Nutrition; NAPs: National Action Plans; NDC: Nationally Determined Contributions; WHO: World Health Organization.

Outcomes	Examples of Actions that Can Be Adopted by the EMR, in line with the Recommendations of the Initiative on Climate Action and Nutrition (I-CAN)
**1. Implementation, action and support**	Support Nationally Determined Contributions (NDC) in the incorporation of climate-resilient and sustainable food systems and nutrition commitments.
	Establish consensus, articulated in a policy paper, on key messages and priority nutrition policy actions in support of climate change adaptation and mitigation measures.
	Reinforce the incorporation of climate-resilient nutrition and food systems in National Action Plans (NAPs).
	Encourage the implementation of nutrition-related measures in NDCs and NAPs.
	Identify major climate-nutrition collaborations and create a community of practice around them.
**2. Capacity building, data and knowledge transfer**	Advocate for more public financing focused on research and development that supports nutrition and climate.
	Provide technical support to countries of the region to facilitate the generation of evidence linking dietary data to environmental metrics, in order to improve evidence-informed decision-making.
	Establish consensus, articulated in a policy paper, on major themes and fundamental nutrition policy actions in support of climate change adaptation and mitigation measures in the region.
	Collaborate with the Global Nutrition Report to highlight successful models of nutrition-promoting climate actions and climate-promoting nutrition measures.
**3. Policy and strategy**	Promote the expansion of climate-smart neglected and underutilized species and fortified/biofortified local crops and staple foods.
	Promote and support the adoption of food-based dietary guidelines that integrate environmental considerations.
	Develop targets for sustainable and healthy consumption, including establishing targets for the consumption of red and processed meat.
	Disseminate technical guidance and encourage implementation of climate-smart food procurement for food in public settings (e.g., school meals and school feeding, health and care facilities).
	Encourage the dissemination of information and campaigns for adopting healthy diets that take into consideration nutrition and climate, especially for children.
	Reinforce the role of national food control systems that adopt the WHO Global Strategy on Food Safety 2022—2030.
**4. Investments**	Collaborate with the Green Climate Fund, the World Bank, the Global Impact Investing Network (GIIN), and the World Benchmark Alliance to incentivize climate-nutrition initiatives, and identify loans that are nutrition and climate-supporting.
	Highlight gaps in funding in relation to climate-smart/nutrition-sensitive investments.

## Data Availability

Not applicable.

## Supplementary Materials

The following supporting information can be downloaded at: https://www.mdpi.com/article/10.3390/ijerph192417086/s1, Table S1: Climate change indicators in the various countries of the EMR. References [203,204,205,206,207,208,209,210,211,212,213,214,215,216,217,218,219,220,221,222,223,224,225,226,227,228,229,230,231,232,233,234,235,236,237,238,239,240,241,242,243,244,245] are cited in Supplementary Materials.

## Abbreviations

Carbon dioxide: CO_2_; EMR: Eastern Mediterranean Region; EMRO: Regional Office for the Eastern Mediterranean; FAO: Food and Agriculture Organization; GCC: Gulf Cooperation Council; GDP: gross domestic products; GHGs: Greenhouse Gases; GIIN: Global Impact Investing Network; GNI: Gross National Income; HIC: high income countries; I-CAN: Initiative on Climate Action and Nutrition; KSA: Kingdom of Saudi Arabia; LBW: low birth weight; LMIC: low and medium income countries; NAPs: National Action Plans; NCDs: non-communicable diseases; ND-GAIN: Notre Dame Global Adaptation Initiative; NDCs: Nationally Determined Contributions; RICCAR: Regional Initiative for the Assessment of Climate Change Impacts on Water Resources and Socio-Economic Vulnerability in the Arab Region; SDGs: Sustainable Development Goals; SNS: Sufficient, nutritious and safe food; UAE: United Arab Emirates; UN: United Nations; UNDP: United Nations Development Programme; UNFCCC: United Nations Framework Convention on Climate Change; UNICEF: United Nations International Children’s Emergency Fund; WHO: World Health Organization.
