# Supplementary material for: Climate Change and Nutrition: Implications for the Eastern Mediterranean Region
Source: Int J Environ Res Public Health. 2022 Dec 19;19(24):17086. doi: 10.3390/ijerph192417086 (PMC9779613; doi:10.3390/ijerph192417086)
Supplement: Supplementary file 1 [file ijerph-19-17086-s001.zip › ijerph-2097157-supplementary.pdf]

Table S1. Climate change indicators in the various countries of the EMR.

	Daily Temp	Extreme Temp/Temp Threshold	Daily Precipitation	Extreme Precipitation	Humidity	CO_2 Emissions
**Afghanistan**	Warming of over 1°C across most regions over the 20th century; an average change of around +1.5°C between the periods 1900–1917, and 2000–2017 [1, 2].<br><br>Mean annual temperature has increased by 0.6˚C since 1960, at an average rate of around 0.13˚C per decade [3].<br>Since 1950, Afghanistan's mean annual temperature has increased significantly and considerably by 1.8°C [4].<br><br>Temperatures also vary greatly by altitude, with mountainous regions	The national average monthly maximum is around 20°C, with July maximum averages around 33°C [2, 3].<br><br>These national averages hide considerable subnational variations and some extremely hot areas such as the cities of Kandahar and Herat, which experience average July maxima of around 40°C and 37°C, respectively [2].<br><br>Annual max temperature was 20.19°C in 1901 vs. 21.9°C in 2021 [5].<br><br>*Warning:*<br>Increased frequency of hot days and nights since 1960, and decreased frequency of cold days and	Historical analysis of precipitation patterns reveals that mean annual quantities have not changed significantly across the country; however, detailed analyses of spring and winter precipitation levels reveal that these changes are simply levelled out as spring precipitation decreased (by up to a third) while winter precipitation slightly increased [4].<br><br>Some changes in precipitation patterns were observed between 1951–2010 across Afghanistan, including slight (<10%) reductions in mean annual rainfall across the west of the country, and less spring rainfall across all regions [2].<br><br>Annual precipitation was 289.63 mm in 1901 vs. 217.9 mm in 2021 [5].	*Drought:*<br>Between 1901 and 2010, there was a significant increase in drought severity in the southern provinces of Kandahar, Helmand and Nimruz during the wheat growing season (November to May), whereas drought intensity during the corn and rice growing seasons (primarily July to September) worsened significantly in the western third of Afghanistan's territory [2, 7].<br><br>Drought events such as that of 2011 have been known to push millions into food insecurity and poverty [2].<br><br>*Flood:*<br>Flood risk is widespread in Afghanistan [2].	-	CO_2 Emissions-World bank [8]:<br>-1990: 2380 kt<br>-2000: 760 kt<br>-2010: 7110 kt<br>-2019: 6080 kt

	experiencing temperatures well below zero on an annual basis, yet southern arid regions regularly experiencing temperatures over 35°C [2].<br><br>Mean annual temperature was 12.75°C in 1901 vs. 14.3°C in 2021 [5].	nights [3]. Warming has been strongest in Afghanistan's central and southwestern regions, and weakest in the northeast in the vicinity of Afghanistan's largest glaciers [2, 6].				
**Bahrain**	Mean air temperature fluctuates between 14°C and 41°C [9].<br><br>Air temperatures have been steadily increasing in Bahrain over the period 1950- 2010. Except for March, temperatures in the 1950s were all at least 0.2°C lower than average monthly temperatures for the entire period. For the 2000s, each month's average	Annual max temperature was 31.79°C in 1901 vs. 33.83°C in 2021 [10].	While mean annual rainfall is 79.7 mm for the period 1950–2008, there is a wide range of spatial and temporal variation between months of the year as well as from year to year. Only in three years which recurred at long intervals did the total annual precipitation exceed 200 mm (intervals of 11 and 19 years). Annual rainfall was lower than 20mm in only six years; these recurred at shorter intervals (between 2 and 10 years) [9].	-	Mean monthly relative humidity is about 67%, with the mean daily maximum ranging from 78% to 88% [9].<br><br>Relative humidity is highest during the winter months of December through February, although other months of the year show only slightly lower levels [11].	CO_2 Emissions-World bank [12]:<br>-1990: 10740 kt<br>-2000: 15880 kt<br>-2010: 25970 kt<br>-2019: 33260 kt

	temperature was at least 0.1°C higher than average monthly temperatures for the entire period [9].<br><br>Mean annual temperature was 27.14°C in 1901 vs. 29.15°C in 2021 [10].		Annual precipitation was 68.83 mm in 1901 vs. 19.79 mm in 2021 [10].			
**Djibouti**	Mean annual mean temperature for Djibouti is 27.8°C, with average monthly temperatures ranging between 23°C (January) and 32°C (July) [13, 14].<br><br>Djibouti experienced temperature increase since the 1970s, with the southern and western regions observing the most significant temperature rise [14].	Mean maximum annual temperature is 32.2°C [13, 14].<br><br>Annual max temperature was 33.01°C in 1901 vs. 33.74°C in 2021 [14].<br><br><u>*Warming:*</u><br>The greatest warming was observed during the summer hot season. A reduction in cool nights and increase in warm nights since 1960 have been observed [13, 15].	Mean annual precipitation is 244.6 mm, with highest rainfall occurring July to September, with relatively very low levels of precipitation occurring nearly all year round [13, 14].<br><br>Annual precipitation was 395.88 mm in 1901 vs. 179.15 mm in 2021 [14].	<u>*Drought:*</u><br>Djibouti has experienced reduced water availability in some areas and increased periods of drought and dry spells; extended drought between 2008 and 2011 [13, 16].<br><br><u>*Flood:*</u><br>Stronger precipitation events resulting in flash flooding in recent years has also been observed due to an increase in heavy precipitation events [13, 16].<br><br><u>*Aridity:*</u><br>Djibouti has also experienced an increase in aridity across the country and intense droughts, the	-	CO_2 Emissions-World bank [17]:<br>-1990: 270 kt<br>-2000: 370 kt<br>-2010: 520 kt<br>-2019: 420 kt

	Mean annual temperature was 27.91°C in 1901 vs. 28.46°C in 2021 [14].			most significant occurring in 1989, 1994, 2004 and 2005 [13, 15].<br><br>*Landslides:*<br>Djibouti has also experienced mudslides and landslides [13].		
**Egypt**	Temperatures range between an average minimum of 14°C in winter and an average maximum of 30°C in summer [18].<br><br>Average annual temperatures are increasing by 0.53°C per decade [19].<br><br>Mean annual temperature was 22.14°C in 1901 vs. 23.88°C in 2021 [20].	Annual max temperature was 29.64°C in 1901 vs. 31.27°C in 2021 [20].	Egypt receives fewer than 80 mm of precipitation annually in most areas. Most rain falls along the coast, but even the wettest area, around Alexandria, receives only about 200 mm of precipitation per year [18].<br><br>Annual precipitation was 25.76 mm in 1901 vs. 21.28 mm in 2021 [20].	*Drought and flood:*<br>Climate change is expected also to increase the occurrence of extreme events like floods and droughts to which all Nile countries are vulnerable [18].<br><br>*Storms:*<br>Sandstorms, often accompanied by winds of up to 140 kilometers per hour, can cause temperatures to rise as much as 20°C in two hours [18].	The city, however, reports humidity as high as 77% during the summer. But during the rest of the year, humidity is low [18].	CO_2 Emissions-World bank [21]:<br>-1990: 87750 kt<br>-2000: 114610 kt<br>-2010: 200310 kt<br>-2019: 249370 kt
**Iran**	Average annual temperatures range between 22°C to 26°C [22].<br><br>Mean annual temperature was 17.64°C in 1901 vs. 19.54°C in 2021 [23].	During the 1975-2020 period, the trend of maximum temperature is 0.03°C /decade; 0.05°C/decade in winter and 0.02°C/decade in summer [22].	Many reports observe a steady decline in annual rainfall (~ 30%) [24].<br><br>The average rainfall in Iran is about 250 mm [22].<br><br>Rain and snow constitute about 70 and 30% of total	*Drought and flood:*<br>The possibility of severe droughts and severe floods will increase in the future [22].<br><br>*Storms:*<br>Central and eastern parts of the country are the	Low humidity in most parts of Iran [22].	CO_2 Emissions-World bank [25]:<br>-1990: 198470 kt<br>-2000: 340450 kt<br>-2010: 541170 kt<br>-2019: 630010 kt

		A significant increasing trend (~ 30%) of hot extremes was revealed in central and southern regions of Iran [24].<br><br>Annual max temperature was 24.79°C in 1901 vs. 26.87°C in 2021 [23].	precipitation. Total precipitation provides 417 bcm water, of which 299 bcm (72%) evaporates [22].<br><br>Between 1975 and 2010, the trend of annual rainfall changes is not significant and is less than -1 mm per decade [22].<br><br>Annual precipitation was 222.66 mm in 1901 vs. 133.63 mm in 2021 [23].	main regions affected by sandstorm. Contrary to the sandstorm, dust storm distributes mostly in western provinces [22].		
**Iraq**	Average annual temperature increase has reached 0.06°C in some areas, while being 0.01°C in others [26].<br><br>Mean annual temperature was 22.21°C in 1901 vs. 24.22°C in 2021 [27].	Summer temperature does not exceed 35°C in most parts [26].<br><br>Annual max temperature was 29.44°C in 1901 vs. 31.68°C in 2021 [27].	Annual rainfalls range between 200 and 400 mm [26].<br><br>Annual rain rate is about 50-200 mm [26].<br><br>Precipitation has been following a decreasing trend (1.2 mm per year) [28].<br><br>Annual precipitation was 205.25 mm in 1901 vs. 148.07 mm in 2021 [27].	*<u>Flood:</u>*<br>Flooding has become more frequent in recent years [29].	-	CO_2 Emissions-World bank [30]:<br>-1990: 64210 kt<br>-2000: 87630 kt<br>-2010: 108550 kt<br>-2019: 174560 kt
**Jordan**	The average temperature across the country varies between 13°C in the southern Badia	The maximum temperature is almost distributed uniformly, across the country, with an average of	Annual precipitation tends to decrease significantly with time at a rate of 1.2mm per year [31].	*<u>Drought:</u>*<br>Increased incidents of drought and evaporation [31].	Humidity tends to increase significantly by an average of 0.08%/year [31].	CO_2 Emissions-World bank [33]:<br>-1990: 9930 kt<br>-2000: 16270 kt<br>-2010: 20200 kt

	region to 28°C at Aqaba, with a mean of 18.6°C all over the country [31].<br><br>The mean, maximum and minimum air temperature tends to increase significantly by 0.02, 0.01, and 0.03 °C/year [31].<br><br>Mean annual temperature was 18.25°C in 1901 vs. 20.36°C in 2021 [32].	25.3°C varying from 18.7 to 31.3°C [31].<br><br>Annual max temperature was 25.87°C in 1901 vs. 27.63°C in 2021 [32].	The annual total precipitation vary sharply, from a minimum of 28 mm at the southern Badia region to a maximum of 570 mm at the upper northern highlands region of Ras Muneef. Along the Ghor region, the highest point located in the north were the annual precipitation it may reach 280 mm at Deir-Alla and decreases towards the south to 71 mm at Ghor Saf [31].<br><br>Annual precipitation was 100.6 mm in 1901 vs. 88.95 mm in 2021 [32].	<u>*Flood:*</u><br>Floods are still a threat, if rainfall events exceed the thresholds. The northern parts of Aqaba are the most vulnerable regions for flashflood hazards [31].		-2019: 24630 kt
**KSA**	The average annual temperature ranges from 11.8°C to 34.5°C in different regions. The average summer temperature inland range is 27° to 45°C while the coastal areas record a range of 27° to 38°C [34].<br><br>The average winter temperature inland ranges from 8° to	The maximum temperature range recorded was 31.2°C in Jazan in July and 46.4°C in Al-Ahsa in June [34].<br><br>Over the period 1978–2019, the maximum temperature increased) (0.60°C per decade). The rate of increase in the maximum temperatures was	Annual precipitation is usually sparse (up to 100 mm or 4 inches in most regions) [34].<br><br>The annual rainfall in Riyadh reported the averages of 100 mm (4 inches) and falls almost exclusively between January and May. The average in Jeddah is 54 mm (2.1 inches) and occurs between November and January [34].	Desertification, i.e. strong winds and dust storms [34].<br><br><u>*Flood:*</u><br>Saudi Arabia has experienced five major floods between 2018 and 2020 that have destroyed properties, loss of plant species, and nutrient depletion [34].	The average humidity range recorded in 2019 was 10% (Madinah and Riyadh in June) and 88% (Arar in January) in different parts of the Kingdom [34].	-

	20°C and 19° to 29°C in coastal areas [34].<br><br>Mean annual temperature was 25.19°C in 1901 vs. 26.8°C in 2021 [35].	reported as 0.71°C per decade, respectively, for the period 1978–2019 [36].<br><br>Annual max temperature was 32.33°C in 1901 vs. 33.92°C in 2021 [35].	Annual precipitation was 103.46 mm in 1901 vs. 85.65 mm in 2021 [35].			
**Kuwait**	The average annual temperature in Kuwait is 26.1°C over the period of 1657-2010 [37]<br><br>In summer months, average daily high temperatures range from 40°C to 44°C [38].<br><br>Mean annual temperature was 25.77°C in 1901 vs. 27.89°C in 2021 [39].	Maximum daily temperatures can reach 45°C in the summer during which there is no rainfall [37, 38].<br><br>Kuwait already is experiencing high temperatures up to 48°C in the summer, with a reading of 54°C in July 2016 north of Kuwait City [38].<br><br>Annual max temperature was 32.73°C in 1901 vs. 34.99°C in 2021 [39].	The average annual rainfall is typically approximately 112 mm per year and varies from 75 to 150 mm/year [38].<br><br>Annual precipitation was 97.3 mm in 1901 vs. 36.81 mm in 2021 [39].	*Drought:*<br>It is a recurrent phenomenon [38].<br><br>*Storms:*<br>Dust storms are particularly frequent in the summer and can reach speeds up to 150 km per hour [38].	From mid-August through September, humidity can exceed 95% [37, 38].<br><br>Relative humidity over the last 48 years (1962–2010) has average about 48%, with mean yearly maximums varying from 84% to 95% [37].<br>Over the period 1987 through 2017, the average relative humidity was 57% [38].	CO_2 Emissions-World bank [40]:<br>-1990: 29130 kt<br>-2000: 49840 kt<br>-2010: 80720 kt<br>-2019: 92650 kt
**Lebanon**	Due to the moderating effect of the sea, the daily temperature range is narrower than it	Summers are hot and humid with temperatures crossing 35°C in August [41-43].	Precipitation in inland Lebanon is higher than precipitation along the coast (1,600 mm) with snow in the mountains [43].	*Storms:*<br>During November and March, rainfall is concentrated during only a few days, falling in	-	CO_2 Emissions-World bank [45]:<br>-1990: 5620 kt<br>-2000: 15670 kt<br>-2010: 20860 kt

	is inland. January is the coldest month, with temperatures around 5 to 10°C [41, 42].<br>Average annual temperature of 15˚C [42, 43].<br><br>Mean temperature has increased at an average rate of 0.15-0.26°C; increasing tread in mean annual temperature is not statistically significant [41].<br><br>Mean annual temperature was 14.22°C in 1901 vs. 16.23°C in 2021 [44].	Annual max temperature was 20.04°C in 1901 vs. 21.69°C in 2021 [44].<br><br>*<u>Warming:</u>*<br>Daily temperature observations show statistically significant trends in the frequency of hot and cold nights [41].	The mean annual rainfall on the coast ranges between 700 and 1,000 mm [42, 43].<br><br>About 70% of the average rainfall in the country falls between November and March and is concentrated during only a few days of the rainy season [41-43].<br><br>Available observations do not show any statistically significant trend in rainfall in Lebanon since 1960 [41].<br><br>The magnitude of annual 1-day rainfall maxima shows statistically significant increases (2.71 mm per decade) between 1960 and 2003 [41].<br><br>Annual precipitation was 470.41 mm in 1901 vs. 610.44 mm in 2021 [44].	heavy cloudbursts or violent storms [43].		-2019: 27950 kt
**Libya**	Mean annual temperature was 21.97°C in 1901 vs. 23.05°C in 2021 [46].	Annual max temperature was 28.77°C in 1901 vs. 29.87°C in 2021 [46].	Annual precipitation was 38.25 mm in 1901 vs. 30.48 mm in 2021 [46].	Libya witnessed a significant decrease in rainfall between October 2020 and October 2021 [47].	-	CO_2 Emissions-World bank [48]:<br>-1990: 28600 kt<br>-2000: 44720 kt<br>-2010: 59550 kt<br>-2019: 56800 kt

**Morocco**	Temperatures in the coastal regions range between 22-25˚C in the summer and 10-12˚C in the winter. The interior border of Morocco experiences string seasonal temperature variations, with average temperatures of 25 to 30˚C in the summer dropping considerably in winter to less than 15˚C in the winter [49].<br><br>Mean annual temperature has increased by 0.9˚C since 1960, an average rate of 0.20˚C per decade [49, 50].	<u>*Warming:*</u><br>The frequency of days that are classed as hot has significantly increased since 1960, while the frequency of cold days and night has significantly decreased [49, 50].	Mean annual rainfall over Morocco has not changed with any consistent trend since 1960 [49].<br><br>An overall decline in precipitation through the past several decades [50].	<u>*Drought:*</u><br>The region is very dry throughout the year [49].<br><br><u>*Flood:*</u><br>Seasonal rainfall patterns have shifted to longer and more intense rain events in October and November, which often cause flooding [50].	-	CO_2 Emissions-World bank [51]:<br>-1990: 21500 kt<br>-2000: 32880 kt<br>-2010: 51760 kt<br>-2019: 71480 kt
**Oman**	Average monthly temperatures typically fluctuate between 10°C to 30°C [52].	The average maximum temperature of the hottest month ranges from 23°C to 42°C. In the interior plain, high temperatures in	While average rainfall is estimated at 9.5 billion m3 per year, about 80% of this precipitation evaporates [52].	<u>*Drought:*</u><br>About 95% of Oman's land area is characterized as a desert or having more than moderate desertification [52].	Humidity can reach more than 90% in coastal regions where it is hot and humid in summer with high	CO_2 Emissions-World bank [54]:<br>-1990: 11920 kt<br>-2000: 25110 kt<br>-2010: 47080 kt<br>-2019: 76030 kt

	On average, annual temperatures have increased in Oman by around 0.4°C per decade [52].<br><br>Mean annual temperature was 27.1°C in 1901 vs. 28.1°C in 2021 [53].	summer can exceed 42°C [52].<br><br>The highest annual average temperatures of 28°C are found along the Sea of Oman coastline between Muscat and Sur [52].<br><br>Annual max temperature was 33.24°C in 1901 vs. 34.25°C in 2021 [53].	Average annual rainfall ranges from 150 mm to 300 mm in the Hajar Mountains in the north while ranging from 50 mm to 150 mm in the Dhofar Mountains in the south. Across the hillsides of these mountain ranges, average annual rainfall is between 100 mm and 150 mm. Over the rest of Oman, annual rainfall is between 0 mm and 50 mm [52].<br><br>Annual precipitation was 60.09 mm in 1901 vs. 31.94 mm in 2021 [53].	Prolonged drought and flooding prevail [52].<br><br>*Storms:*<br>Tropical cyclonic storms and severe cyclonic storms have tracked toward Oman over the past decades [52].	temperatures of 40°C [52].	
**Pakistan**	Warming in Pakistan was estimated at 0.57°C over the 20th century. Warming has accelerated more recently, with 0.47°C of warming measured between 1961–2007. On a sub-national level, warming is strongly biased towards the more southerly regions, with Punjab, Sind, and	There are places where temperature rises as high as 50-54°C in summers [58].<br><br>The rise in average daily maximum temperatures (0.87°C between 1961–2007) has been slightly stronger than the rise in average temperatures [55].<br><br>Many regions of Pakistan experience	The early 20th century was characterised by a prolonged decline in annual rainfall, but since 1960, a slight increasing trend has prevailed. Mean rainfall in the arid plains of Pakistan and the coastal belt has decreased by 10%–15% since 1960 [55, 59].<br><br>Pakistan receives rainfall of less than 250 mm annually, except in the southern slopes of Himalaya and the sub mountainous regions in	*Drought:*<br>At present Pakistan faces an annual median probability of severe meteorological drought of around 3% [55, 61]. Pakistan has experienced serious drought situation from 1998-2002 facing adverse freshwater dearth [58].<br><br>*Flood:*<br>Pakistan also has high exposure to flooding (ranked jointly 8th),	Summer season is extremely hot and the humidity ranges between 25% to 50% [58].	CO_2 Emissions-World bank [62]:<br>-1990: 59030 kt<br>-2000: 98370 kt<br>-2010: 140620 kt<br>-2019: 190570 kt

Balochistan all experiencing winter warming in the region of 0.91°C–1.12°C over the same period, and Khyber Pakhtunkhwa in the north experiencing only 0.52°C [55].<br><br>Mean annual temperature has increased by 0.35˚C since 1960, an average rate of 0.08˚C per decade [56].<br><br>Warming between the periods 1900–1917 and 2000–2017, showed 1.3°C of warming in the vicinity of Islamabad compared to 0.9°C of warming in the vicinity of Karachi [55].<br><br>Mean annual temperature was	temperatures of 38°C and above on an annual basis [55].<br><br>Annual max temperature was 27.59°C in 1901 vs. 28.82°C in 2021 [57].<br><br>*Warming and heat wave:*<br>Pakistan regularly experiences some of the highest maximum temperatures in the world, with an average monthly maximum of around 27°C and an average June maximum of 36°C [55].<br>A concurrent increase in the frequency of heat wave days has been documented, particularly in Sindh Province [55, 59].<br>Historical warming has been strongest in western Pakistan [55].<br>Pakistan experienced 126 heat waves between 1997–2015, around 7 per year,	the northern segment of the country, where annual rainfall ranges from 760 mm to 2,000 mm [58].<br><br>Mean annual rainfall over Pakistan has not changed with any discernible trend since 1960 [56].<br><br>Annual precipitation was 245.7 mm in 1901 vs. 228.18 mm in 2021 [57].	including, riverine, flash, and coastal [55].<br>For example, following the hugely devastating flood of 2010, Pakistan experienced back to back floods every year during 2011-2015 [58].<br><br>*Landslides:*<br>Pakistan has also experienced landslides between 1900 and 2020 [55].

	20.51°C in 1901 vs. 21.68°C in 2021 [57].	with an increasing trend [55, 60].<br><br>The frequency of hot days an d nights has increased significantly since 1960, while the frequency of cold days and nights has decreased [56].				
**Palestine**	The mean summer temperatures range from 30°C in Jericho through 25°C in Gaza to 22°C in Hebron [63].<br>The mean temperatures range in winter from 13°C in Jericho and Gaza to 7°C in Hebron [63].<br><br>An analysis of daily temperature data from 1976 to 1995 has shown an increase in mean temperature of 0.4°C [64, 65].	-	Average rainfall for the West Bank during 2008-2009 was 425 mm – 22% below the historic average of 538 mm, and at 316.3 mm for the Gaza Strip - 12% below the historic average [65].	Urban areas are sensitive to floods, heat waves, droughts, and other extreme events [63].	-	-
**Qatar**	Annual mean temperature increased by 0.3°C	Mean maximum temperature reaches 41.3°C in summer [66].	Annual rainfall was 82 mm during 1990-2008; with the rainfall being 30% greater	*__Flood:__*<br>It was estimated that Qatar is susceptible to	-	CO_2 Emissions-World bank [68]:<br>-1990: 12540 kt<br>-2000: 28660 kt

	over the last 40 years [66].<br><br>Mean annual temperature was 27.5°C in 1901 vs. 29.42°C in 2021 [67].	Annual max temperature was 32.66°C in 1901 vs. 34.59°C in 2021 [67].	in the Northern part of the country [66].<br><br>Annual precipitation was 70.05 mm in 1901 vs. 19.18 mm in 2021 [67].	inland flooding with 18.2% of its land area [66].		-2010: 60580 kt<br>-2019: 91970 kt
**Somalia**	Annual mean temperature is 27°C [69].<br><br>The country has experienced a gradual and continuous increase of 1°C to 1.5°C in median annual temperatures since 1991 [70].<br><br>Mean annual temperature was 26.62°C in 1901 vs. 26.88°C in 2021 [71].	The highest mean maximum value during the period 1963–1990) is 42°C in June and July at Berbera [69].<br><br>Median daily maximum temperatures in Somalia range from 30°C to 40°C [70].<br><br>Annual max temperature was 32.03°C in 1901 vs. 32.32°C in 2021 [71].	Somalia experiences low and erratic precipitation with an average annual rainfall of 250 mm [69].<br><br>The northern part of the country is characterized by extremely hot and arid conditions with an average rain of less than 250 mm, while the average annual rainfall in the south is approximately 400 mm and 700 mm in the south-west. In the central semi-arid parts, the rainfall received is as low as 50-100 mm/year [69].<br><br>Annual precipitation was 301.67 mm in 1901 vs. 300.36 mm in 2021 [71].	<u>*Drought and flood:*</u><br>Droughts occur every 2-3 years [69].<br><br>The amount of rainfall received across Somalia varies dramatically from year to year with recurrent drought periods that persist for several years, and erratic periods of intense downpours and flooding [69].	Relatively high humidity across the whole country [69].	-
**Sudan**	Temperatures have been increasing between 0.2°C and 0.4°C per decade [72, 73].	Annual max temperature was 35.61°C in 1901 vs. 35.89°C in 2021 [74].	Rainfall has been increasing between 20 mm and 30 mm per decade in the northernmost and	<u>*Drought:*</u><br>Drought is now one of the most important and frequently recurring challenges that Sudan	Relative humidity ranges between 42-70% [72].	CO_2 Emissions-World bank [75]:<br>-1990: 5370 kt<br>-2000: 5690 kt<br>-2010: 16420 kt

	Mean annual temperature was 27.74°C in 1901 vs. 28.17°C in 2021 [74].		southernmost areas of the territory [72].<br><br>Mean annual rainfall ranges between 33 mm/year in Hala'ib in the north to about 73 mm/year in Tokar in the south [72].<br><br>Annual precipitation was 225.75 mm in 1901 vs. 262.75 mm in 2021 [74].	faces. Since the end of the last drought in 1984, droughts have recurred in 1987, 1989, 1990, 1991, 1993, and 1996, mainly in western Sudan in Kordofan and Darfur states, as well as in areas in central Sudan [72].<br><br>*Flood:*<br>The frequency of floods has been noticeably increasing [72].		-2019: 20620 kt
**Syria**	The annual average temperature is 18.1°C on the coastal plain and 15.2°C in the mountains [76].<br><br>Mean annual temperature was 17.94°C in 1901 vs. 19.72°C in 2021 [77].	The mean maximum temperature in August (the hottest month) is 29.9°C on the coastal plain and 27.4°C in the mountains. At some locations in the coastal plains were recorded absolute maximum temperatures of 40.1 – 42°C [76].<br><br>Annual max temperature was 25.17°C in 1901 vs. 26.73°C in 2021 [77].	The average of total precipitations in the coastal plain is about 800 mm per annum more than 1,200 mm per annum in the mountains, about 300-500 mm per annum in western inland and northeastern areas, above 600 mm in the Golan heights and the western part of the inland mountains, about 100-250 mm and less in the steppe (al-Badia), and 200-250 mm in its marginal zone (transition into dry farming areas) [76].	*Drought:*<br>Considered one of the main challenges affecting Syria's development [76]. Starting in 2006, however, and lasting into 2011, Syria experienced a multiseason, multiyear period of extreme drought [78, 79].<br><br>*Heat waves and storms:*<br>Heat waves and dust storms are on the increase [76].	In the coastal area relative humidity is high in summer, reaching 73%, and even higher on hot days in the plains. It drops in the mountains by about 8%; its yearly mean is about 67% [76].	CO_2 Emissions-World bank [80]:<br>-1990: 32390 kt<br>-2000: 44400 kt<br>-2010: 61090 kt<br>-2019: 25710 kt

			Annual precipitation was 215.66 mm in 1901 vs. 232.68 mm in 2021 [77].			
**Tunisia**	Annual average temperatures for the country range between 16°C and 20°C, with hot summers. Tunisia's winters are relatively mild with average temperatures between 10°C and 14°C [81, 82].<br><br>Mean annual mean temperature for Tunisia is 19.4°C, with average monthly temperatures ranging between 28°C (June, July) and 10°C (January) [82].<br><br>Tunisia has experienced a significant increase in its temperatures over the last 30 years, with temperatures	Summer mean temperatures often exceed 25°C with max temperatures reaching 32°C [81, 82].<br><br>Annual max temperature was 25.2°C in 1901 vs. 27.07°C in 2021 [84].<br><br>*Warming:*<br>Greater warming has been observed during the summer season (May to September), rather than the winter. A reduction in cool nights and increase in warm nights since 1960 have been observed [82, 85].	Annual rainfall has decreased 5% per decade in the northern part of Tunisia since 1950, while heavy rainfall events have become more frequent [83].<br><br>The majority of rain falls along the relatively humid, coastal areas, with southern areas receiving as little as 150 mm per year [82].<br><br>Mean annual precipitation is 263.5 mm, with highest rainfall occurring September to April, with relatively very low levels of precipitation occurring nearly all year round [82, 84].<br><br>Annual precipitation was 282.57 mm in 1901 vs. 145.53 mm in 2021 [84].	Precipitation was observed to have decreased approximately 3% over the past 30 years [82].<br><br>*Drought:*<br>Tunisia has also experienced increased periods of drought and dry spells; in 1982, 1987 to 1989, 1993 to 1995, and its worst drought in over 50 years from 1999 to 2002 [82, 83, 85].<br><br>*Flood:*<br>Stronger precipitation events resulting in flash flooding in recent years has also been observed [82, 85].<br><br>*Aridity:*<br>Tunisia has also experienced increasingly frequent occurrences of aridity in the recent years [82].<br><br>*Landslides:*	-	CO_2 Emissions-World bank [86]:<br>-1990: 14540 kt<br>-2000: 20700 kt<br>-2010: 27520 kt<br>-2019: 29910 kt

	increasing by 0.37°C per decade. Tunisia has observed a mean average temperature increase of 1.4°C since 1901 [82, 83].<br><br>Mean annual temperature was 19.06°C in 1901 vs. 21.33°C in 2021 [84].			Tunisia has also experienced landslides [82].		
**UAE**	Average temperature is about 26˚C during the daytime and 15˚C during the nighttime [87].<br><br>Mean annual temperature was 27.75°C in 1901 vs. 29.08°C in 2021 [88].	Temperatures can rise to about 48˚C in coastal cities [87].<br><br>In the southern desert regions temperatures can climb to 50°C with very low humidity [87].<br><br>Annual max temperature was 34.19°C in 1901 vs. 35.54°C in 2021 [88].	The country averages between 140- 200 mm of rainfall per year, with some mountainous areas experiencing up to 350 mm/year [87].<br><br>Annual precipitation was 66.65 mm in 1901 vs. 51.46 mm in 2021 [88].	<u>*Storms:*</u><br>Prone to occasional and violent dust storms, known as shamal winds [87].	Humidity levels reaching as high as 90% [87].	CO_2 Emissions-World bank [89]:<br>-1990: 55210 kt<br>-2000: 84730 kt<br>-2010: 162790 kt<br>-2019: 188860 kt
**Yemen**	Mean temperatures in the highlands range from below 15°C in winter to 25°C in summer, and in the coastal lowlands from 22.5°C in winter to	Annual max temperature was 31.37°C in 1901 vs. 31.88°C in 2021 [94].<br><br><u>*Warming:*</u><br>Warming of about 1.4°C per century since	Annual average precipitation varies between 50 mm in the lower parts near Al-Kawd and 650 mm in the upper part of wadi near Yarim. Throughout the basin,	<u>*Drought:*</u><br>Yemen has experienced periods of drought [90]; a severe drought during early 2013 [92].<br><br><u>*Flood:*</u>	High relative humidity (i.e., reaching 93%) [92].	-

up to 35°C in summer [90-92].<br><br>The average annual temperature over the 1961-1990 period is about 20.4°C, with the highest monthly temperature of 23.7°C occurring in July [92].<br><br>Annual mean temperatures have risen across Yemen since the 1960s [90, 93]. Mean annual temperature has increased by 1.8˚C since 1960, a rate of around 0.39˚C per decade [91].<br><br>Mean annual temperature was 25.12°C in 1901 vs. 25.62°C in 2021 [94].	the 1900s. On average across this country, this warming has been 0.5°C over the 20th century [90, 93].	rainfall average about nearly 380 mm/year [92].<br><br>Rainfall regimes differ in the highlands and in coastal areas, with relatively little rainfall received in the center of the country. Coastal areas receive 80% of the annual rainfall during the winter months [90, 95].<br><br>Since the 1950s, summer precipitation totals appear to have declined across the Yemen Highlands, at a rate of 1.2 mm per month (9%) per decade [90, 91].<br><br>Annual precipitation was 201.69 mm in 1901 vs. 186.75 mm in 2021 [94].	Rainfall in Yemen is characterized by seasonally intense and short-lived heavy storms that often lead to flash floods [90].<br><br>*Landslides:*<br>The entire country is at risk of landslides. A vast majority of the urban poor is vulnerable to rockslide and landslide risk as they live in informal settlements that are typically on marginal and environmentally sensitive land [90].

Abbreviations: Carbon dioxide: CO_2; EMR: Eastern Mediterranean Region; KSA: Kingdom of Saudi Arabia; UAE: United Arab Emirates.

References

1. Carbon brief. Mapped: How every part of the world has warmed – and could continue to. Infographics. Available online: https://www.carbonbrief.org/mapped-how-every-part-of-the-world-has-warmed-and-could-continue-to-warm/ (accessed on 4 November 2022).
2. World Bank Group and Asian Development Bank. *Climate risk country profile - Afghanistan*; 2021; Available online: https://openknowledge.worldbank.org/bitstream/handle/10986/36381/Afghanistan-Climate-Risk-Country-Profile.pdf?sequence=1&isAllowed=y.
3. McSweeney, C.; New, M.; Lizcano, G. *UNDP climate change country profile - Afghanistan*; 2012; Available online: https://www.geog.ox.ac.uk/research/climate/projects/undp-cp/index.html?country=Afghanistan&d1=Reports.
4. Islamic Republic of Afghanistan. *Second National Communication under the UNFCCC*; 2017; Available online: https://www4.unfccc.int/sites/SubmissionsStaging/NationalReports/Documents/9486351_Afghanistan-NC2-1-SNC%20Report_Final_20180801%20.pdf.
5. World Bank Climate Change Knowledge Portal. Climate change overview: Afghanistan. Available online: https://climateknowledgeportal.worldbank.org/country/afghanistan (accessed on 8 November 2022).
6. Aich, V.; Akhundzadah, N. A.; Knuerr, A.; Khoshbeen, A. J.; Hattermann, F.; Paeth, H.; Scanlon, A.; Paton, E. N. Climate change in Afghanistan deduced from reanalysis and coordinated regional climate downscaling experiment (CORDEX)—South Asia simulations. *Climate* **2017,** *5*, 38.
7. Qutbudin, I.; Shiru, M. S.; Sharafati, A.; Ahmed, K.; Al-Ansari, N.; Yaseen, Z. M.; Shahid, S.; Wang, X. Seasonal drought pattern changes due to climate variability: Case study in Afghanistan. *Water* **2019,** *11*, 1096.
8. World Bank. Climate change: CO2 emissions - Afghanistan. Available online: https://data.worldbank.org/topic/climate-change?locations=AF (accessed on 4 November 2022).
9. Kingdom of Bahrain. *Bahrain's second national communication under the UNFCCC*; 2012; Available online: https://unfccc.int/resource/docs/natc/bhrnc2.pdf.
10. World Bank Climate Change Knowledge Portal. Climate change overview: Bahrain. Available online: https://climateknowledgeportal.worldbank.org/country/bahrain (accessed on 8 November 2022).
11. Kingdom of Bahrain. *Bahrain's Third National Communication under the United Nations Framework Convention on Climate Change*; 2020; Available online: https://unfccc.int/sites/default/files/resource/9143680_Bahrain-NC3-2-SCE%20Third%20National%20Communication%202020.pdf.
12. World Bank. Climate change: CO2 emissions - Bahrain. Available online: https://data.worldbank.org/topic/climate-change?locations=BH (accessed on 4 November 2022).
13. World Bank Group. *Climate risk country profile - Djibouti*; 2021; Available online: https://climateknowledgeportal.worldbank.org/sites/default/files/2021-04/15722-WB_Djibouti%20Country%20Profile-WEB.pdf.
14. World Bank Climate Change Knowledge Portal. Djibouti. Available online: https://climateknowledgeportal.worldbank.org/country/djibouti/climate-data-historical (accessed on 4 November 2022).

15. Republic of Djibouti. *Second National Communication to the UNFCCC*; 2014; Available online: https://unfccc.int/sites/default/files/resource/djinc2.pdf.
16. Kireyev, M. A. P. Macro-fiscal implications of climate change: the case of Djibouti. **2018**.
17. World Bank. Climate change: CO2 emissions - Djibouti. Available online: https://data.worldbank.org/topic/climate-change?locations=DJ (accessed on 4 November 2022).
18. Egyptian Environmental Affairs Agency. *Egypt Third National Communication under the United Nations Framework Convention on Climate Change*; 2016; Available online: https://unfccc.int/sites/default/files/resource/TNC%20report.pdf.
19. UNICEF. Climate change - Egypt. Available online: https://www.unicef.org/egypt/climate-change (accessed on 7 November 2022).
20. World Bank Climate Change Knowledge Portal. Climate change overview: Egypt. Available online: https://climateknowledgeportal.worldbank.org/country/egypt (accessed on 8 November 2022).
21. World Bank. Climate change: CO2 emissions - Egypt. Available online: https://data.worldbank.org/topic/climate-change?locations=EG (accessed on 4 November 2022).
22. Islamic Republic of Iran. *Third National Communication to United Nations Framework Concention on Climate Change (UNFCCC)*; 2017; Available online: https://unfccc.int/sites/default/files/resource/Third%20National%20communication%20IRAN.pdf.
23. World Bank climate Change Knowledge Portal. Climate change overview: Islamic Republic of Iran. Available online: https://climateknowledgeportal.worldbank.org/country/iran-islamic-rep (accessed on 8 November 2022).
24. Mansouri Daneshvar, M. R.; Ebrahimi, M.; Nejadsoleymani, H. An overview of climate change in Iran: facts and statistics. *Environ Syst Res* **2019,** *8*, 1-10.
25. World Bank. Climate change: CO2 emissions - Iran. Available online: https://data.worldbank.org/topic/climate-change?locations=IR (accessed on 4 November 2022).
26. Republic of Iraq. *Iraq's Initial National Communication to the UNFCCC*; 2016; Available online: https://unfccc.int/sites/default/files/resource/316947520_Iraq-NC1-2-INC-Iraq.pdf.
27. World bank climate Change Knowledge Portal. Climate change overview: Iraq. Available online: https://climateknowledgeportal.worldbank.org/country/iraq (accessed on 8 November 2022).
28. Food and Agriculture Organization of the United Nations. *Building resilience to cope with climate change in Jordan through improving water use efficinecy in the agriculture sector (BRCCJ)*; 2020; Available online: https://jordan.un.org/en/111315-building-resilience-cope-climate-change-jordan-through-improving-water-use-efficiency.
29. United Nations. Migration, Environment, and Climate Change in Iraq. Available online: https://iraq.un.org/en/194355-migration-environment-and-climate-change-iraq (accessed on 7 November 2022).
30. World Bank. Climate change: CO2 emissions - Iraq. Available online: https://data.worldbank.org/topic/climate-change?locations=IQ (accessed on 4 November 2022).
31. Hashemite Kingdom of Jordan. *Jordan's Third National Communication on Climate Change*; 2014; Available online: https://unfccc.int/resource/docs/natc/jornc3.pdf.

32. World Bank Climate Change Knowledge Portal. Climate change overview: Jordan. Available online: https://climateknowledgeportal.worldbank.org/country/jordan (accessed on 8 November 2022).
33. World Bank. Climate change: CO2 emissions - Jordan. Available online: https://data.worldbank.org/topic/climate-change?locations=JO (accessed on 4 November 2022).
34. Designate National Authority. *Fourth National Communication - UNFCCC*; 2022; Available online: https://unfccc.int/sites/default/files/resource/7123846_Saudi%20Arabia-NC4-1-Fourth%20National%20Communication%20NC4%20Kingdom%20of%20Saudi%20Arabia%20March%202022.pdf.
35. World Bank Climate Change Knowledge Portal. Climate change overview: Saudi Arabia. Available online: https://climateknowledgeportal.worldbank.org/country/saudi-arabia (accessed on 8 November 2022).
36. Almazroui, M. Changes in temperature trends and extremes over Saudi Arabia for the period 1978–2019. *Adv Meteorol* **2020,** *2020*.
37. State of Kuwait-Environment Public Authority. *Kuwait's Initial National Communications under the United Nations Framework Convention on Climate Change*; 2012; Available online: https://unfccc.int/resource/docs/natc/kwtnc1.pdf.
38. Environment of Public Authority-Kuwait. *State of Kuwait Second National Communication - UNFCCC*; 2019; Available online: https://unfccc.int/sites/default/files/resource/94235106_Kuwait-NC2-2-KUWAIT%20SNC%20%20final%20v2.pdf.
39. World Bank Climate Change Knowledge Portal. Climate change overview: Kuwait. Available online: https://climateknowledgeportal.worldbank.org/country/kuwait (accessed on 8 November 2022).
40. World Bank. Climate change: CO2 emissions - Kuwait. Available online: https://data.worldbank.org/topic/climate-change?locations=KW (accessed on 4 November 2022).
41. Karmalkar, A.; McSweeney, C.; New, M.; Lizcano, G. *UNDP climate change country profile - Lebanon*; 2012; Available online: https://www.geog.ox.ac.uk/research/climate/projects/undp-cp/index.html?country=Lebanon&d1=Reports.
42. Ministry of Environment; United Nations Development Programme; Global Environment Facility. *Lebanon's Fourth Biennial update report the UNFCCC*; 2021; Available online: https://unfccc.int/sites/default/files/resource/Lebanon%20BUR4%202021.pdf.
43. Ministry of Environment-Lebanon. *Lebanon's Third National Communication to the UNFCCC*; 2016; Available online: https://unfccc.int/sites/default/files/resource/lbnnc3.pdf.
44. World Bank climate Change Knowledge Portal. Climate change overview: Lebanon. Available online: https://climateknowledgeportal.worldbank.org/country/lebanon (accessed on 8 November 2022).
45. World Bank. Climate change: CO2 emissions - Lebanon. Available online: https://data.worldbank.org/topic/climate-change?locations=LB (accessed on 4 November 2022).
46. World Bank Climate Change Knowledge Portal. Climate change overview: Libya. Available online: https://climateknowledgeportal.worldbank.org/country/libya (accessed on 8 November 2022).
47. OCHA. *Libya: Conflict weakens farmers' abilities to mitigate climate risks*; 2022; Available online: https://reliefweb.int/report/libya/libya-conflict-weakens-farmers-abilities-mitigate-climate-risks-enar.
48. World Bank. Climate change: CO2 emissions - Libya. Available online: https://data.worldbank.org/topic/climate-change?locations=LY (accessed on 4 November 2022).

49. McSweeney, C.; New, M.; Lizcano, G. *UNDP climate change country profile - Morocco*; 2012; Available online: https://www.geog.ox.ac.uk/research/climate/projects/undp-cp/index.html?country=Morocco&d1=Reports.
50. The World Bank. *Morocco Country Climate and Development Report*; 2022; Available online: https://www.worldbank.org/en/country/morocco/publication/morocco-country-climate-and-development-report.
51. World Bank. Climate change: CO2 emissions - Morocco. Available online: https://data.worldbank.org/topic/climate-change?locations=MA (accessed on 4 November 2022).
52. Ministry of Environment and Climate Affairs-Oman. *Second National Communication*; 2019; Available online: https://unfccc.int/sites/default/files/resource/Oman%202nd%20National%20Communication%20%2817%20November%202019%29%20-%20Final.pdf.
53. World Bank Climate Change Knowledge Portal. Climate change overview: Oman. Available online: https://climateknowledgeportal.worldbank.org/country/oman (accessed on 8 November 2022).
54. World Bank. Climate change: CO2 emissions - Oman. Available online: https://data.worldbank.org/topic/climate-change?locations=OM (accessed on 4 November 2022).
55. World Bank Group and Asian Development Bank. *Climate risk country profile - Pakistan*; 2021; Available online: https://openknowledge.worldbank.org/bitstream/handle/10986/36372/Pakistan-Climate-Risk-Country-Profile.pdf?sequence=1&isAllowed=y.
56. McSweeney, C.; New, M.; Lizcano, G. *UNDP climate change country profile - Pakistan*; 2012; Available online: https://www.geog.ox.ac.uk/research/climate/projects/undp-cp/index.html?country=Pakistan&d1=Reports.
57. World Bank Climate Change Knowledge Portal. Climate change overview: Pakistan. Available online: https://climateknowledgeportal.worldbank.org/country/pakistan (accessed on 8 November 2022).
58. Ministry of Climate Change-Government of Pakistan. *Pakistan's Second National Communication on Climate Change to United Nations Framework Convention on Climate Change*; 2018; Available online: http://www.gcisc.org.pk/SNC_Pakistan.pdf.
59. Asian Development Bank. *Climate change profile of Pakistan*; 2017; Available online: https://www.adb.org/publications/climate-change-profile-pakistan.
60. Nasim, W.; Amin, A.; Fahad, S.; Awais, M.; Khan, N.; Mubeen, M.; Wahid, A.; Rehman, M. H.; Ihsan, M. Z.; Ahmad, S. Future risk assessment by estimating historical heat wave trends with projected heat accumulation using SimCLIM climate model in Pakistan. *Atmos Res* **2018,** *205*, 118-133.
61. World Bank Climate Change Knowledge Portal. Climate projections - Pakistan. Available online: https://climateknowledgeportal.worldbank.org/country/pakistan/climate-data-projections (accessed on 4 November 2022).
62. World Bank. Climate change: CO2 emissions - Pakistan. Available online: https://data.worldbank.org/topic/climate-change?locations=PK (accessed on 4 November 2022).
63. Environment Quality Authority-State of Palestine. *Initial National Communication Report to the United Nations Framework Convention on Climate Change*; 2016; Available online: https://unfccc.int/sites/default/files/resource/Initial%20National%20Communication%20Report_%20State%20of%20Palestine.pdf.
64. El-Kadi, A. K. Global warming: a study of the Gaza temperature variations in the period 1976-1995. *J Hum Res* **2005,** *13*.

65. UNDP. *Climate Change Adaptation Strategy and Programme of Action for the Palestinian Authority*; 2013; Available online: https://www.undp.org/papp/publications/palestinian-climate-change-adaptation-strategy.
66. Ministry of Environment-State of Qatar. *Initial National Communication to the United Nations Framework Convention on Climate Change*; 2011; Available online: https://unfccc.int/sites/default/files/resource/final%20climate%20change.pdf.
67. World Bank Climate Change Knowledge Portal. Climate change overview: Qatar. Available online: https://climateknowledgeportal.worldbank.org/country/qatar (accessed on 8 November 2022).
68. World Bank. Climate change: CO2 emissions - Qatar. Available online: https://data.worldbank.org/topic/climate-change?locations=QA (accessed on 4 November 2022).
69. The Federal Republic of Somalia. *The Initial National Communication for Somalia to the United Nations Framework Convention on Climate Change*; 2018; Available online: https://www4.unfccc.int/sites/SubmissionsStaging/NationalReports/Documents/91826345_Somalia-NC1-1-Somalia%20Final%20%20FNC%20Final.pdf.
70. OCHA. *The impact of climate change on peace and security in Somalia*; 2021; Available online: https://reliefweb.int/report/somalia/impact-climate-change-peace-and-security-somalia.
71. World Bank Climate Change Knowledge Portal. Climate change overview: Somalia. Available online: https://climateknowledgeportal.worldbank.org/country/somalia (accessed on 8 November 202).
72. Sudan, R. o. t. *Sudan's Second National Communication under the United Nations Framework Convention on Climate Change*; 2013; Available online: https://unfccc.int/resource/docs/natc/sudnc2.pdf.
73. Funk, C.; Eilerts, G.; Verdin, J.; Rowland, J.; Marshall, M. *A climate trend analysis of Sudan. U.S. Geological Survey Fact Sheet 2011*; 2011; Available online: https://pubs.usgs.gov/fs/2011/3072/.
74. World Bank Climate Change Knowledge Portal. Climate change overview: Sudan. Available online: https://climateknowledgeportal.worldbank.org/country/sudan (accessed on 8 November 2022).
75. World Bank. Climate change: CO2 emissions - Sudan. Available online: https://data.worldbank.org/topic/climate-change?locations=SD (accessed on 4 November 2022).
76. Ministry of State for Environment Affairs; UNDP; Global Environmental Facility. *Initial National Communication of the Syrian Arab Republic - UNFCCC*; 2010; Available online: https://unfccc.int/resource/docs/natc/syrnc1.pdf.
77. World Bank climate Change Knowledge Portal. Climate change overview: Syrian Arab Republic. Available online: https://climateknowledgeportal.worldbank.org/country/syrian-arab-republic (accessed on 8 November 2022).
78. Gleick, P. H. Water, drought, climate change, and conflict in Syria. *Weather Clim Soc* **2014,** *6*, 331-340.
79. Worth, R. Earth is parched where Syrian farms thrived. Available online: https://www.nytimes.com/2010/10/14/world/middleeast/14syria.html#:~:text=Syria%20continues%20to%20grow%20cotton,against%20foreign%20dependence%2C%20analysts%20say. (accessed on
80. World Bank. Climate change: CO2 emissions - Syria. Available online: https://data.worldbank.org/topic/climate-change?locations=SY (accessed on 4 November 2022).

81. Tunisia. *Tunisia's third national communication as part of the UNFCCC*; 2019; Available online: https://unfccc.int/sites/default/files/resource/Synth%C3%A8se%20Ang%20Finalis%C3%A9.pdf.
82. World Bank Group. *Climate risk country profile - Tunisia*; 2021; Available online: https://climateknowledgeportal.worldbank.org/sites/default/files/2021-02/15727-WB_Tunisia%20Country%20Profile-WEB.pdf.
83. OCHA. *Climate Change Profile: Tunisia*; 2019; Available online: https://reliefweb.int/report/tunisia/climate-change-profile-tunisia.
84. World Bank Climate Change Knowledge Portal. Tunisia. Available online: https://climateknowledgeportal.worldbank.org/country/tunisia/climate-data-historical (accessed on 4 November 2022).
85. GERICS. Climate fact sheet - Tunisia. Available online: https://www.climate-service-center.de/products_and_publications/fact_sheets/climate_fact_sheets/index.php.en (accessed on 4 November 2022).
86. World Bank. Climate change: CO2 emissions - Tunisia. Available online: https://data.worldbank.org/topic/climate-change?locations=TN (accessed on 4 November 2022).
87. Ministry of Energy-UAE. *Third National Communication under the United Nations Framework Convention on Climate Change*; 2012; Available online: file:///C:/Users/mt86/Downloads/United%2520Arab%2520Emirates.pdf.
88. World Bank Climate Change Knowledge Portal. Climate change overview: United Arab Emirates. Available online: https://climateknowledgeportal.worldbank.org/country/united-arab-emirates (accessed on 8 November 2022).
89. World Bank. Climate change: CO2 emissions - UAE. Available online: https://data.worldbank.org/topic/climate-change?locations=AE (accessed on 4 November 2022).
90. Global Facility for Disaster Reduction and Recovery. *Climate risk and adaptation country profile. Vulnerability, risk reduction and adaptation to climate change - Yemen*; 2011; Available online: https://www.gfdrr.org/en/publication/climate-risk-and-adaptation-country-profile-yemen.
91. McSweeney, C.; New, M.; Lizcano, G. *UNDP climate change country profile - Yemen*; 2012; Available online: https://www.geog.ox.ac.uk/research/climate/projects/undp-cp/index.html?country=Yemen&d1=Reports.
92. Environment Protection Authority-Yemen. *Third National Communication to the Conference of the Parties of United Nations Framework Convention on Climate Change*; 2018; Available online: https://unfccc.int/sites/default/files/resource/Yemen_TNC_%202018.pdf.
93. Mitchell, T. D.; Hulme, M.; New, M. Climate data for political areas. *Area* **2002,** *34*, 109-112.
94. World Bank Climate Change Knowledge Portal. Climate change overview: Republic of Yemen. Available online: https://climateknowledgeportal.worldbank.org/country/yemen-rep (accessed on 8 November 2022).
95. World Bank. *Assessing the Impact of Climate Change and Variability on the Water and Agriculture Sectors, and the Policy Implications - Yemen*; 2010; Available online: https://openknowledge.worldbank.org/bitstream/handle/10986/2943/541960ESW0Gray1OFFICIAL0USE0ONLY191.pdf?sequence=1&isAllowed=y.
